# The Role of BaCO_3_ on Ni-BaZrO_3_ Catalysts for Efficient NH_3_ Decomposition

**DOI:** 10.1021/acsomega.6c08031

**Published:** 2026-10-13

**Authors:** Andrea Felli, Maila Danielis, Christine Artner-Wallner, Giovanni Capurso, Alessandro Trovarelli, Sara Colussi

**Affiliations:** † Polytechnic Department, 9316 University of Udine, Via del Cotonificio 108, 33100 Udine, Italy; ‡ 344665 Treibacher Industrie AG, Auer-von-Welsbach-Straße 1, 9330 Althofen, Austria

## Abstract

Barium zirconate (BaZrO_3_) is a versatile material that has recently gained attention as a catalyst support for ammonia decomposition. In this study, an innovative synthesis strategy for BaZrO_3_ is combined with a solvent-free dry-milling method to deposit nickel onto its surface. The resulting Ni-BaZrO_3_ catalyst exhibited superior performance in ammonia decomposition for CO_*x*_-free hydrogen production, outperforming both traditionally synthesized BaZrO_3_-based systems and more conventional Ni-supported catalysts. Comprehensive material characterization—using XRD, N_2_ adsorption–desorption, ATR, and Raman spectroscopy—confirmed that the new synthesis route improves the structural and chemical properties of the perovskite compared to conventional methods. H_2_-TPR and CO_2_-TPD analyses revealed that exploiting superficial BaCO_3_ reduction to BaO enhanced surface defectivity, and a high density of basic sites interacting with Ni nanoparticles can be obtained, facilitating electron exchange between the support and the metal. The mechanism of BaO formation and interaction with CO_2_ in TPD was deeply analyzed using *in situ* characterization techniques. Further mechanistic insights into product formation and release were obtained via TPSR and DRIFTS, emphasizing the critical role of metal-support interaction through the formation of Ni-BaO entities on the catalyst surface. Overall, this work demonstrates a sustainable and scalable route for producing high-performance Ni-BaZrO_3_ catalysts and advances the understanding of their reactivity, focusing on the importance of the metal-support interplay and reinforcing their potential in green hydrogen production through ammonia decomposition.

## Graphical abstract

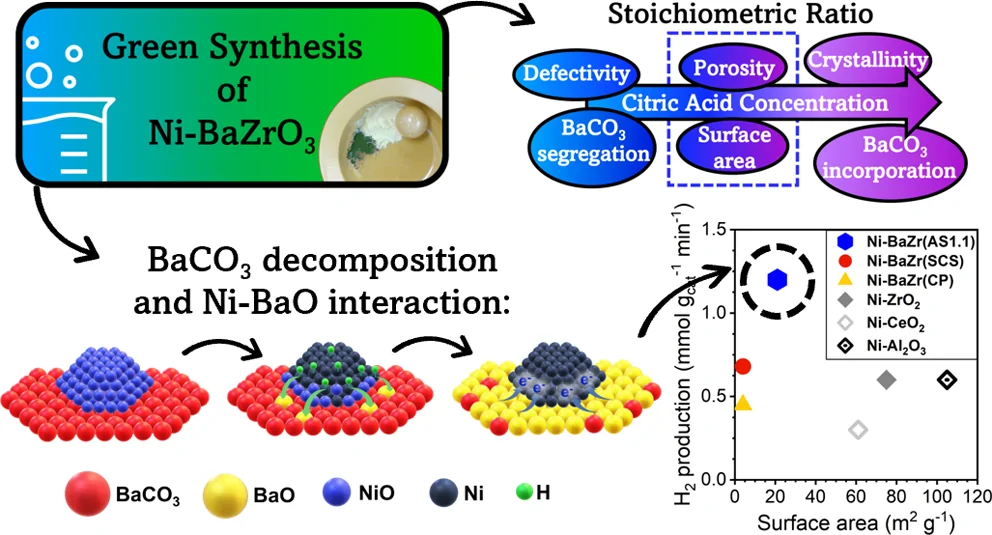

## 1 Introduction

Large-scale hydrogen storage and transportation are challenged by hydrogen intrinsic properties, particularly its low volumetric energy density and broad flammability range, which raise technical and safety concerns.^1^ Among the proposed alternatives, ammonia has recently gained attention as a promising hydrogen carrier due to its high hydrogen content (17.3 wt %), ease of liquefaction at mild conditions (7.5 bar at 20 °C), and compatibility with existing infrastructure.^2^ Unlike hydrogen, ammonia can be stored and transported more efficiently, avoiding the need for high-pressure or cryogenic systems. Moreover, its decomposition yields hydrogen without CO_*x*_ emissions.^3,4^

Hydrogen release from ammonia is achieved through thermocatalytic decomposition, but the process requires highly active catalysts to be energy-efficient and industrially viable. Although Ru-based catalysts remain the most effective, their high cost, limited availability, and environmentally harmful extraction processes have driven interest toward more sustainable alternatives.^4,5^ As a result, increasing research efforts are focused on earth-abundant transition metals such as Fe, Co, and Ni.^6,7^ Equally important is the identification of optimal supports to enhance the performance of these non-noble-metal catalysts and potentially match or surpass the activity of Ru-based systems.

In this context, PGM-free perovskites (ABO_3_) are gaining significant and growing interest as catalysts for ammonia cracking.^8–12^ Among them, BaZrO_3_ has recently emerged as a promising support for Ni- and Ru-based catalysts for ammonia decomposition.^8,11,13^ DFT and microkinetic studies show that barium zirconate is particularly effective combined with Ni due to its high basicity and the presence of surface vacancies that promote NH_3_ dehydrogenation at the metal-support interface.^14^ Nevertheless, a thorough search of the relevant literature yielded only one experimental paper investigating Ni-BaZrO_3_ for ammonia decomposition,^8^ whereas other papers considered bimetallic Ni–Co supported on BaZrO_3_ ^11^ and Ni supported on yttrium-doped BaZrO_3_.^15^ Recently, Ren et al. compared the catalytic activity of K–Ni/BaZrO_3_ with that of K-RuNi/BaZrO_3_, mainly focusing on the properties of the resulting bimetallic alloy rather than on the role of the supporting perovskite.^16^ Thus, a comprehensive study of the factors influencing its catalytic activity and strategies to enhance it remains lacking but is extremely important, also considering that BaZrO_3_ is a protonic conductor^17^ and its relevance expands to ammonia-fed protonic ceramic fuel cells.^18–20^ One of the main drawbacks is the low surface area usually obtained for perovskites via conventional synthesis methods. Moreover, traditional synthesis routes, including self-combustion and coprecipitation, typically involve the use of nitrate precursors, corrosive chemicals, and organic solvents, raising environmental and safety concerns due to the emission of greenhouse gases, the use of hazardous substances, and the generation of open flames,^21,22^ issues that should be avoided when aiming at industrial scalability of the process.

Several synthesis methods have been developed to investigate the effects of doping, particle size distribution, sintering behavior, and mechanical properties of BaZrO_3_.^17,23,24^ In these studies, parameters critical for catalysis—such as surface area, defect density, and basicity—are often overlooked. Another critical factor affecting BaZrO_3_ is the formation and stabilization of BaCO_3_ during synthesis. BaCO_3_ tends to form when Ba is exposed to air or when organic precursors, complexing agents, surfactants, or chelating agents are used.^22^ As an insulating compound, BaCO_3_ hinders the sintering process, leading to the common practice of calcining BaZrO_3_ at high temperatures (>1100 °C) to remove carbonate residues and obtain a pure perovskite phase.^25^ However, such conditions can degrade the surface properties, increase synthesis costs and duration, and promote Ba volatilization, which may significantly alter the material performance.^26^ Despite BaCO_3_ being generally viewed as an undesired phase, no conclusive evidence has been reported linking its presence to a detrimental effect on the catalytic activity of BaZrO_3_-based catalysts. Carbonate species on the material surface can instead have a positive role in catalysis, as recently demonstrated for Ni-BaCO_3_ catalyst^27^ and AZrO_3_ (A = Na, Mg, Ca, Sr, Ba) perovskites,^28^ where they can be advantageously exploited to capture CO_2_ and convert it into methane through chemical-looping processes. Moreover, the decomposition of BaCO_3_ on Co/MgO catalysts has already been reported as beneficial for NH_3_ synthesis and decomposition reactions.^29,30^

In this work, an innovative synthetic strategy for the preparation of high-performance Ni-BaZrO_3_ catalysts for ammonia decomposition is introduced. A novel modified citrate method was developed to prepare BaZrO_3_ supports that were subsequently loaded with 10 wt % Ni using a low-energy, environmentally friendly, mechanochemical approach. The entire synthesis process was designed to minimize environmental impact, reduce costs, and enable industrial scalability. The obtained materials were compared to Ni-BaZrO_3_ counterparts synthesized via conventional routes. The impact of BaCO_3_ segregation from the BaZrO_3_ perovskite on the catalytic activity for NH_3_ decomposition was investigated in depth for the first time, highlighting its role on the formation of BaO sites interacting with Ni when treating the materials in a reducing environment. The interfacial interaction of Ni-BaO is discussed in relation to the catalytic performance of Ni-BaZrO_3_. Several key aspects of the Ni-BaZrO_3_ catalytic system were thus systematically studied by combining different experimental techniques, providing new insights into its behavior and advancing the development of efficient, PGM-free catalysts for ammonia cracking.

## 2 Results and Discussion

### 2.1 Morphological Properties of Synthesized BaZrO_3_ and Ni-BaZrO_3_

Table 1 reports the sample names, the corresponding citric acid-to-cation ratio CA/C^+^ used, and the summary of all the relevant data from the chemical and morphological characterization of BaZrO_3_ supports and Ni-BaZrO_3_ catalysts. XRF analysis of all samples confirmed the Ba:Zr = 1 molar stoichiometry, which corresponds to the weight ratio BaO/ZrO_2_ = 1.22.

**Table 1:** Summary of Sample Names, Elemental Composition, and Textural Properties of Synthesized BaZrO_3_ and Ni-BaZrO_3_

sample name	CA/C^+^	BaO/(ZrO_2_ + HfO_2_)^a^	BET SSA (m^2^ g^–1^)	avg. pore volume (cm^3^ g^–1^)^b^	avg. pore size (nm)^b^	avg. crystalline size (nm)^c^
BaZr(AS0.4)	0.4	1.23	8	0.011	9.4	23
BaZr(AS0.6)	0.6	1.25	9	0.040	12.8	24
BaZr(AS0.8)	0.8	1.21	10	0.042	15.2	35
BaZr(AS1.0)	1.0	1.23	17	0.093	21.4	21
BaZr(AS1.1)	1.1	1.22	21	0.095	17.8	22
BaZr(AS1.2)	1.2	1.23	16	0.091	18.5	32
BaZr(AS1.3)	1.3	1.19	11	0.048	14.5	28
BaZr(AS1.5)	1.5	1.23	10	0.045	15.9	23
BaZr(AS1.8)	1.8	1.22	15	0.063	13.3	20
BaZr(AS2.0)	2.0	1.23	9	0.039	14.6	23
BaZr(CP)	–	1.22	4	0.020	17.9	48
BaZr(SCS)	1.1	1.21	5	0.039	21.9	38
NiBaZr(AS1.1)	1.1	1.22	20	0.091	17.3	22
NiBaZr(CP)	–	1.22	4	0.018	17.5	48
NiBaZr(SCS)	1.1	1.21	4	0.037	21.9	38

a Measured by XRF analysis.

b Obtained from the desorption branch of N_2_ adsorption/desorption isotherm.

c Calculated by Scherrer’s equation.

Figure 1a shows the specific surface area (SSA) of BaZrO_3_ as a function of citric acid concentration, while isotherm curves for selected samples are provided in Figure S1. BaZr­(AS) samples exhibit significantly higher SSA values than BaZr­(SCS) and BaZr­(CP). SSA gradually increases for CA/C^+^ ratios between 0.4 and 0.8, then sharply rises to 21 m^2^ g^–1^ at CA/C^+^ = 1.1, with a second maximum observed at CA/C^+^ = 1.8. This dual-peak trend, occasionally reported in the literature,^31^ highlights the importance of optimizing CA concentration. Under substoichiometric conditions (CA/C^+^ < 1), SSA remains below 10 m^2^ g^–1^ due to insufficient complexation during gel formation,^21,23^ while a further increase in CA (CA/C^+^ > 1.2) leads to gel densification and reduced SSA. The highest SSA values are obtained in the range of CA/C^+^ = 1.0–1.2, where optimal coordination is achieved. Average pore size and volume follow a trend similar to that of SSA (Figure 1b). Pore sizes range from 9.4 to 21.4 nm, peaking near the stoichiometric CA/C^+^ ratio. Pore size distributions for representative samples are shown in Figure S2. Compared to SCS and CP materials, AS samples exhibit a well-defined mesoporous structure (3–80 nm), with AS1.1 showing the highest porosity. These results indicate that stoichiometric CA/C^+^ conditions favor the formation of a mesoporous structure. Overall, the AS consistently yields materials with higher SSA and well-developed mesoporosity, confirming the effectiveness of the synthesis approach, particularly for applications in catalysis.

**Figure 1:**
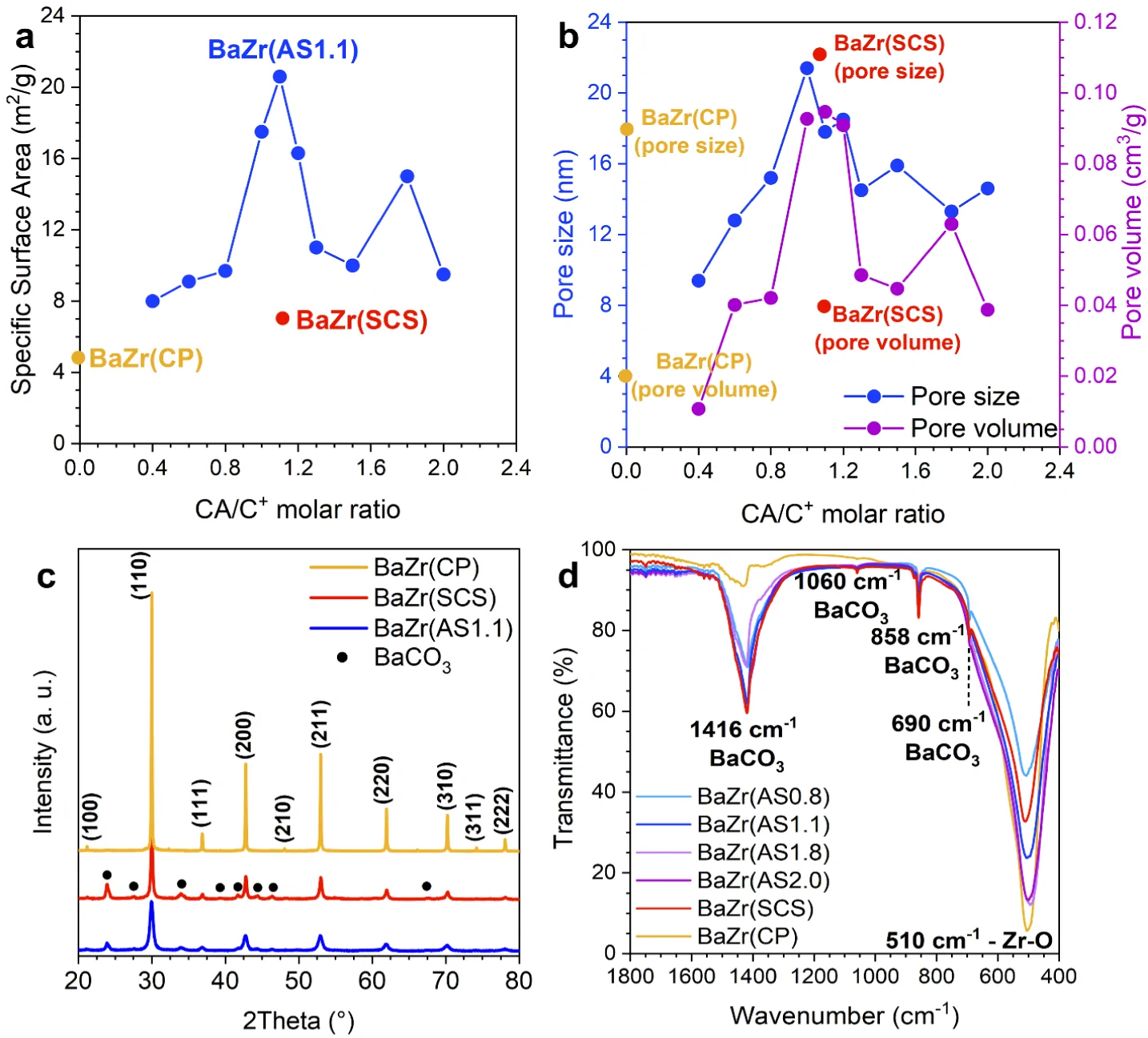
(a) Specific surface area and (b) average pore size and average pore volume of BaZrO_3_ supports as a function of the citric acid-to-cation ratio (CA/C^+^); (c) X-ray diffractograms of BaZr­(CP), BaZr­(SCS), and BaZr­(AS1.1); (d) ATR-IR spectra of BaZrO_3_ samples.

Thus, among the AS samples, BaZr­(AS1.1) was selected as representative and used as the catalyst support for NH_3_ decomposition due to its high SSA and porosity. NiBaZr­(AS1.1) was prepared and compared with NiBaZr­(SCS) and NiBaZr­(CP). As shown in Table 1, the SSA and pore size of the Ni-BaZrO_3_ catalysts are largely determined by the original support, with the mechanochemical Ni deposition having minimal impact. The pore size distribution profiles of BaZr­(AS1.1) and NiBaZr­(AS1.1) remain nearly identical (Figure S3), indicating that the porous structure is retained after milling, as expected by using these mild milling conditions.^32^

XRD analysis was performed to confirm the formation of the BaZrO_3_ perovskite phase. Figure 1c shows the diffractograms of BaZr­(AS1.1), BaZr­(SCS), and BaZr­(CP). The coprecipitated sample displays well-defined reflections corresponding to the cubic BaZrO_3_ phase (Pm-3m, ICSD No. 97460)^33^ and exhibits high crystallinity with no secondary phases. In contrast, BaZr­(AS1.1) and BaZr­(SCS) show lower crystallinity and the presence of BaCO_3_, consistent with the relatively low calcination temperature (800 °C) and the synthesis conditions.^28^ The influence of the CA/C^+^ ratio on crystallinity was further examined (Figures S4–S5). At substoichiometric CA/C^+^ ratios (e.g., BaZr­(AS0.8)), reduced crystallinity and significant BaCO_3_ segregation were observed, likely due to incomplete complexation during gel formation. Increasing the CA concentration over the stoichiometric levels, instead, improved crystallinity and reduced BaCO_3_ content. Table 1 reports the crystallite sizes for all samples calculated via the Scherrer equation, ranging from 20 nm for BaZr­(AS1.8) to 35 nm for BaZr­(AS0.8). Overall, the AS method produces smaller crystallites (average 25 nm) compared to CP (48 nm) and SCS (38 nm), reflecting the SSA trend and further confirming the effectiveness of the modified synthesis route. XRD patterns of the Ni-BaZrO_3_ catalysts are shown in Figure S6. No distinct peaks related to Ni phases are observed, likely due to the formation of NiO during calcination, whose diffraction signals (JCPDS No. 04–0835)^34^ overlap with those of the BaZrO_3_ perovskite, preventing the estimation of Ni dispersion and/or particle size from the diffractograms. NiO formation can be anyway deduced by TGA experiments, as reported in Figure S7 and discussed in the Supporting Information.

ATR and Raman spectroscopy were employed to further examine BaCO_3_ segregation and the crystallinity of the synthesized materials, providing complementary insights to the XRD analysis. ATR results are shown in Figure 1d, while detailed Raman peak assignments and discussion are provided in the Supporting Information (see Figure S8 and Table S1). ATR reveals several bands in the 1800–400 cm^–1^ range. The band near 510 cm^–1^ corresponds to the Zr–O stretching vibration in the [ZrO_6_] octahedra of the cubic perovskite lattice,^21^ which is more intense in highly crystalline samples due to reduced defectivity and high symmetry. Four additional bands between 1800 and 600 cm^–1^ are attributed to BaCO_3_ species,^35^ arising from vibrational modes of CO_3_^2–^ anions: in-plane vibration at 690 cm^–1^, out-of-plane bending at 858 cm^–1^, symmetric stretching at 1059 cm^–1^, and asymmetric stretching at 1416 cm^–1^. The intensity of these carbonate-related peaks decreases with increasing CA/C^+^ ratio, consistently with XRD results showing higher crystallinity and reduced BaCO_3_ segregation at higher CA/C^+^ values. Additionally, Raman spectra (Figure S8c) show that BaZr­(AS) samples exhibit enhanced surface and bulk defectivity due to [ZrO_6_] distortion, BaCO_3_ segregation and displaced oxygen atoms, with decreasing defect concentration as CA/C^+^ increases. Nevertheless, BaZr­(AS1.1) still clearly shows bands at 311 cm^–1^ (Zr antiparallel displacement),^36^ F_2g_(O) vibration at 457 cm^–1^, and E_u_(O) modes at 500 and 640 cm^–1^, linked to bulk and surface displaced oxygen atoms,^36,37^ differently from the other samples where these bands are absent or barely visible.

In conclusion, the AS method led to the formation of a perovskite phase accompanied by Ba segregation as BaCO_3_. Compared to the CP and the SCS samples, the obtained materials exhibited a lower crystallinity but higher SSA, improved porosity, and higher defectivity. By varying the CA/C^+^ ratio, it was possible to tune the surface area, pore structure, crystallinity, and extent of the BaCO_3_ segregation. Among all samples, BaZr­(AS1.1) showed the best compromise, combining the highest SSA with moderate BaCO_3_ segregation and relevant defectivity, and was therefore selected as a Ni support for catalytic ammonia decomposition.

### 2.2 Catalytic Ammonia Decomposition Activity

The catalytic performance of Ni-BaZrO_3_ catalysts is presented as light-off curves in Figure 2a and the corresponding calculated reaction rates at 380 °C are reported in Figure 2b. All the materials were pretreated in H_2_ at 550 °C as described in Methods.

**Figure 2:**
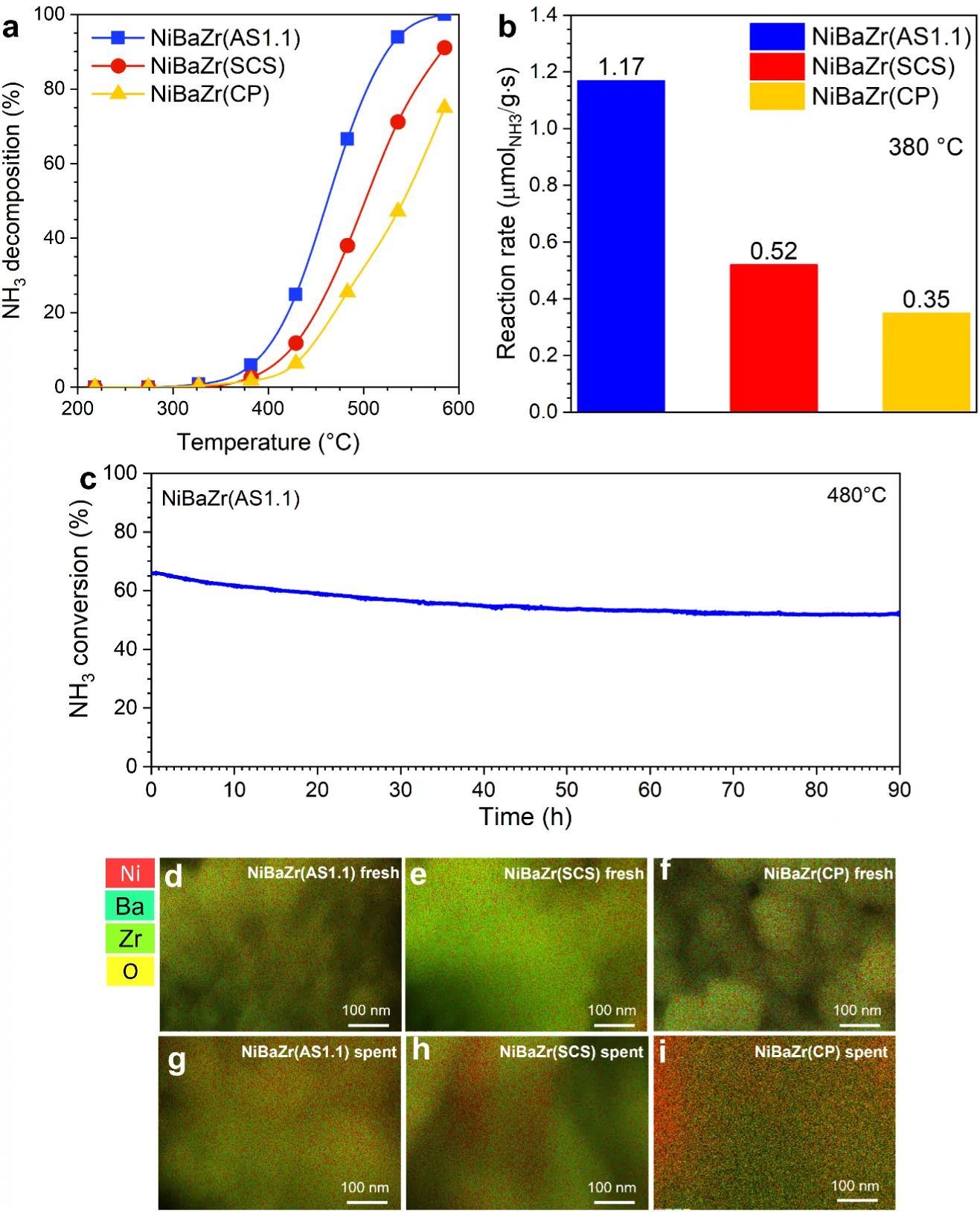
(a) Light-off curves of Ni-BaZrO_3_ catalysts for the ammonia decomposition reaction (GHSV = 30,000 h^–1^); (b) reaction rates at 380 °C of Ni-BaZrO_3_ catalysts; (c) stability test of 90 h on the NiBaZr­(AS1.1) catalyst; (d-f) EDX maps of fresh samples; (g-i) EDX maps of spent samples.

Among the three samples, NiBaZr­(AS1.1) exhibits the highest activity, with a reaction rate of 1.17 μmol g^–1^ s^–1^ at 380 °C, which is more than double compared to the other samples (0.52 μmol g^–1^ s^–1^ for SCS and 0.35 μmol g^–1^ s^–1^ for CP, respectively), and achieving full ammonia conversion at 570 °C, unlike the SCS and CP counterparts. Additionally, a 90 h time-on-stream test was conducted on NiBaZr­(AS1.1) to assess its stability (Figure 2c). The catalyst exhibited a total activity loss of only approximately 10%, with deactivation occurring mostly within the first 30 h, followed by a stable behavior.

To understand the potential of NiBaZr­(AS1.1) with respect to state-of-the-art supported nickel catalysts, Table S2 compares NiBaZr­(AS1.1) with similar systems and other representative literature benchmarks. For the other Ni-BaZrO_3_ catalyst reported in the literature, with a much higher nickel loading (40 wt %) and a much lower space velocity (6000 mL g^–1^ h^–1^), the conversion is 95% at 550 °C.^8^ A similar conversion is attained on 30% Ni supported on 20%Ba/YSZ,^15^ whereas for a bimetallic Ni–Co/BaZrO_3_ sample (40%Ni–Co in a Ni:Co ratio of 9:1), the conversion at the same temperature is 20%.^11^ All these experiments were carried out at much higher nickel loadings and in 100% NH_3_, somehow affecting a straightforward comparison. The experimental conditions used by Ren et al. are instead similar to those used in the present study, leading to a NH_3_ conversion of 56% at 450 °C despite the lower Ni loading.^16^ This fact could be ascribed to the high Ni dispersion on the perovskite surface as well as to the presence of K. Hence, a further comparison with reference catalytic compositions was performed internally with other Ni-based catalysts (Ni-CeO_2_, Ni-ZrO_2_, Ni-Al_2_O_3_, named NiCe, NiZr, and NiAl, respectively) prepared by milling and in the same testing conditions. SSA values and reaction rates are reported in Table S3, while light-off curves are shown in Figure S9. Tables S2 and S3 show that NiBaZr­(AS1.1) has indeed promising catalytic performances among samples tested in more similar conditions. Moreover, from the benchmark comparison data, NiBaZr­(AS1.1) achieved nearly double the H_2_ production rate compared to the other catalysts (shown in Figure S10, where the H_2_ production at 480 °C is reported as a function of the SSA), suggesting that a synergistic effect is established between Ni and the optimized structural and surface properties of BaZrO_3_ obtained through the acetate synthesis route.

In a first analysis, this superior performance could be attributed to the higher SSA and porosity of BaZr­(AS1.1), together with higher defectivity inferred from Raman spectroscopy (Figure S8c). The SCS-based catalyst, with low crystallinity and BaCO_3_ segregation similar to NiBaZr­(AS1.1), also outperforms the CP sample, highlighting the limitations of coprecipitation for BaZrO_3_-based materials for catalytic applications, even when high phase purity is achieved.

XRD analysis of spent catalysts (i.e., after reaction) also allowed an estimation of the average Ni particle size. The spent NiBaZr­(AS1.1) shows an average Ni particle size of 34 nm, smaller than those observed in NiBaZr­(SCS) (41 nm) and NiBaZr­(CP) (46 nm), as calculated from the Ni(111) XRD peak (Figure S11). The smaller Ni particle size observed on NiBaZr­(AS1.1) is consistent with an increased metal dispersion and a larger number of accessible metallic sites for NH_3_ decomposition, ultimately promoting higher catalytic activity. The formation of these smaller Ni nanoparticles is reasonably associated with the higher SSA of NiBaZr­(AS1.1), which enhances the deposition efficiency of Ni acetate during milling and results in a more uniform and finely dispersed Ni phase, even after the 90 h stability test. In fact, XRD analysis of the spent samples (Figure S12) showed sharper Ni peaks after the time-on-stream NH_3_ exposure, indicating Ni sintering. Yet, the calculated average Ni particle size only slightly increases, from 34 to 38 nm. SEM-EDX analyses were further performed to assess the homogeneity of Ni dispersion on both fresh (i.e., before reaction) and spent samples. As shown in Figure 2d–f, no significant differences were observed among the fresh catalysts, and Ni appeared uniformly distributed across all materials. In contrast, the EDX characterization of the spent samples (Figure 2g–i) revealed marked differences. While NiBaZr­(AS1.1) retained a homogeneous Ni distribution within the analyzed area, both NiBaZr­(SCS) and NiBaZr­(CP) exhibited evidence of Ni agglomeration and an overall loss of dispersion. This behavior can be attributed to the lower SSA of these two samples, which promotes Ni particle sintering and crystallite growth during reaction.

Regarding the effect of Ni particle size on catalytic ammonia cracking, in the literature, there is a general agreement that below ∼15 nm the highest activity is achieved for small nanoparticles, <5 nm.^5,38,39^ This enhancement is commonly attributed to the formation of highly reactive surface configurations, such as steps, edges, and B_5_-type sites, on very small Ni nanoparticles.^5,38^ This could explain the activity of the 5%Ni on BaZrO_3_ catalyst reported in Table S2, where the average Ni particle size is ∼2.5 nm.^16^ For larger particle sizes, the situation is less straightforward and, interestingly, in the previous work focusing on 40%Ni on BaZrO_3_, Ni clusters are the largest when supported on BaZrO_3_ (∼100 nm), yet showing the highest catalytic activity among different perovskite supports.^8^ This indicates that for the Ni-BaZrO_3_ system other factors, besides particle size, are likely involved in its catalytic performance. In fact, this is expressed by the increase in activity achieved by decreasing the Ni particle size from ca. 34 nm Ni particles, as reported here, to 2.5 nm, as reported by Ren et al.,^16^ where the activity does not proportionally improve with the strong reduction in average particle size.

### 2.3 Redox and Surface Properties

To further investigate the effects of BaZrO_3_ chemical properties on the reactivity, H_2_-TPR and CO_2_-TPD analyses were performed. Figure 3a shows the TPR profiles of BaZrO_3_ supports and Ni-BaZrO_3_ catalysts. BaZr­(AS1.1) and BaZr­(SCS) exhibit similar hydrogen uptake peaks at ∼300 °C, ∼500 °C, and ∼800 °C. In contrast, BaZr­(CP) shows a more pronounced low-temperature peak at ∼350 °C and an anticipated high-temperature peak at ∼750 °C. While the literature commonly attributes these signals to the reduction of adsorbed species (low-T), surface oxygen (mid-T), and lattice oxygen (high-T),^40,41^ gas evolution data collected during TPR (Figure 3b) indicate that the observed peaks are primarily related to the decomposition of BaCO_3_. CO_2_ evolution occurs between 400 and 600 °C, followed by significant CO release starting around 450 °C, with two distinct peaks at ∼550 °C and ∼800 °C. This CO release correlates with H_2_ consumption in a nearly 1:1 ratio, suggesting the following reaction 1:

1 $${\mathrm{BaCO}}_{3\left(s\right)}+H_{2\left(g\right)}\to {\mathrm{BaO}}_{\left(s\right)}+{\mathrm{CO}}_{\left(g\right)}+H_{2}O_{\left(g\right)}$$

**Figure 3:**
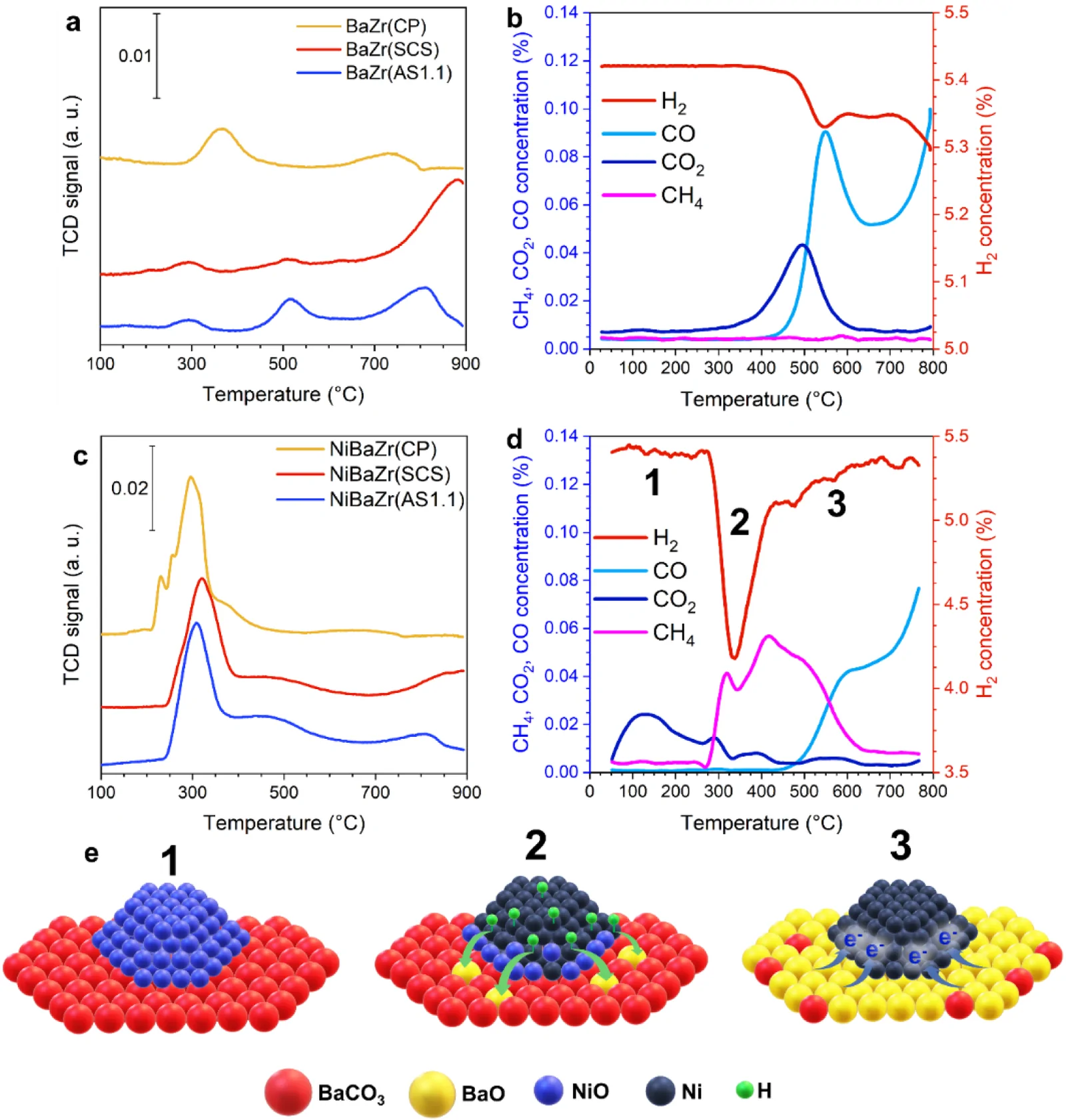
(a) H_2_-TPR analysis of BaZr­(CP), BaZr­(SCS), and BaZr­(AS1.1) supports; (b) gas evolution during BaZr­(AS1.1) reduction; (c) H_2_-TPR analysis of NiBaZr­(CP), NiBaZr­(SCS), and NiBaZr­(AS1.1) catalysts; (d) gas evolution during NiBaZr­(AS1.1) reduction; (e) graphical representation of the proposed reduction mechanism of Ni-BaZrO_3_ samples.

Additionally, the presence of two distinct forms of BaCO_3_ can be inferred: a surface species that decomposes between 400 and 600 °C, releasing CO_2_ and CO, and a bulk species that decomposes at higher temperatures (above 700 °C), primarily into CO. The H_2_-driven decomposition of surface BaCO_3_ generates BaO and, possibly, surface oxygen vacancies,^42^ which have been shown by DFT calculations to promote NH_3_ dehydrogenation on Ni-BaZrO_3_ catalysts.^14^ Due to its higher SSA and smaller particle size, BaZr­(AS1.1) contains a larger fraction of surface BaCO_3_ and a smaller fraction of bulk BaCO_3_ compared to BaZr­(SCS).

Nickel has a strong influence on the decomposition of barium carbonate under hydrogen atmosphere, and their interplay can produce interesting surface structures with strong Ni-BaCO_3_ interaction.^27^ TPR profiles of Ni-BaZrO_3_ catalysts (Figure 3c) show a similar behavior for NiBaZr­(AS1.1) and NiBaZr­(SCS), while NiBaZr­(CP) presents different features. On NiBaZr­(CP), the reduction of surface species^43^ appears clearly as two distinct peaks at 230 °C and 255 °C, whereas on NiBaZr­(AS1.1) and NiBaZr­(SCS), reduction begins around 250 °C, likely delayed due to carbonate presence. The main peak at ∼300 °C is attributed to NiO particles weakly interacting with the support,^44^ while the shoulder between 400 and 500 °C corresponds to NiO in strong metal-support interaction,^45,46^ that is stronger on NiBaZr­(AS1.1) and NiBaZr­(SCS) compared to NiBaZr­(CP), which is another factor that can explain the higher catalytic activity of AS1.1 and SCS samples with respect to CP. TPR profiles of the other benchmark catalysts (Figure S13) also confirm stronger Ni–support interaction in AS and SCS catalysts compared to the other samples—especially NiCe—as shown by the broad hydrogen consumption between 400 and 500 °C. Despite their lower SSAs, NiBaZr­(AS1.1) and NiBaZr­(SCS) both outperformed the NiCe, NiZr, and NiAl samples (Table S3 and Figures S9 and S10).

On BaZrO_3_, the reduction of NiO is accompanied by the decomposition of BaCO_3_, which has been followed by monitoring gas evolution during the reduction of NiBaZr­(AS1.1) (Figure 3d). According to the experimental evidence, the hypothesized reduction mechanism is schematically represented in Figure 3e. Initially, adsorbed CO_2_ is released (Step 1), leaving the surface covered with BaCO_3_. As temperature rises, CH_4_ is produced alongside the reduction of weakly bound NiO. Methane production can result from BaCO_3_ decomposition and subsequent CO/CO_2_ methanation catalyzed by metallic Ni^27^ and/or by the direct BaCO_3_ hydrogenation on the surface by hydrogen spillover from Ni nanoparticles, with a mechanism already suggested for CaCO_3_ ^47^ (Step 2), according to reaction 2:

2 $${\mathrm{BaCO}}_{3}+4H_{2}\to \mathrm{BaO}+{\mathrm{CH}}_{4}+2H_{2}O$$

From 500 °C, CO evolves—likely from the reverse water gas shift reaction and further BaCO_3_ decomposition—while CH_4_ declines. By ∼550 °C, BaCO_3_ is largely decomposed, forming surface BaO and completing NiO reduction (Step 3). It is worth noting that the simultaneous evolution of methane and CO_2_ in the range 280–600 °C is suggestive of the formation of oxygen vacancies on the surface. As further proof, the quantitative analysis of gaseous species consumed and produced is reported in Table S4. Interestingly, by considering all the possible reactions involved, an excess of H_2_ consumption was observed for both the BaZr­(AS1.1) and NiBaZr­(AS1.1) samples, suggesting the reduction of surface oxygen and, therefore, the formation of oxygen vacancies and defects, strongly promoted by the presence of Ni. Thus, a mild (5% H_2_) reducing pretreatment at 550 °C is sufficient to induce the decomposition of surface BaCO_3_, facilitated by the close proximity between Ni and BaCO_3_ species, ultimately leading to the formation of a Ni-BaO interfacial structure and surface vacancies. Indeed, Zhang et al. observed the migration of Ba species from BaCO_3_ to form an overlayer on Ni nanoparticles under hydrogen atmosphere. This restructuring is driven by the decomposition of BaCO_3_ promoted by the presence of Ni and generating a strong metal-support interaction.^27^ Similarly, Sato et al. demonstrated that reduction treatments applied to Co/Ba/MgO catalysts induce a rearrangement of BaO species on the surface of the metal nanoparticles leading to the formation of characteristic surface morphologies that depend on the pretreatment temperature.^29,30^ The intimate contact between BaO and the metal results in the partial encapsulation of metal nanoparticles within a BaO matrix promoting electron donation from BaO to adjacent Co atoms, consequently weakening the triple bonds of N_2_ adsorbed on metal surface.^30^ Analyzing the TPR results in light of these previous evidences, it is reasonable to assume that on NiBaZr­(AS1.1), the formation of BaO, promoted by the contact of Ni with BaCO_3_ and creating a strong metal-support interaction, can enhance the electron donation from the support to Ni, enriching the metal electron density that in turn can facilitate N_2_ and H_2_ recombination and release during NH_3_ decomposition. These results indicate that surface-segregated BaCO_3_ near Ni particles can act as precursors for the formation of an interface between Ni nanoparticles and BaO basic sites, upon proper activation, ultimately enhancing ammonia cracking activity.

Since surface basicity plays a key role in ammonia decomposition and it is usually widely investigated as a fundamental property for ammonia cracking catalysts,^4,5^ CO_2_-TPD experiments were conducted to assess it on supports and on Ni-loaded catalysts. As shown in Figure 4a, CO_2_-TPD profiles of BaZr­(AS1.1) vary by changing the temperature of the reducing pretreatment (reduction with 5% H_2_ in He, 40 mL min^–1^, see SI). Fresh BaZr­(AS1.1) shows a desorption peak at low temperature due to surface-adsorbed CO_2_, and a major desorption between 400 and 750 °C (maximum at 600 °C) likely due to surface BaCO_3_ decomposition, in good agreement with TPR experiments. After reducing pretreatment at 350 °C, the profile remains similar, indicating BaCO_3_ is still intact. However, increasing the reduction temperature up to 550 °C, the 400–750 °C peak disappears, replaced by a weaker 350 °C peak originating from medium basic sites. These results show that CO_2_ desorption in the 400–750 °C range on the fresh sample is not linked to real basic sites, but it is instead related to BaCO_3_ decomposition that, in turn, exposes new basic defect sites capable of CO_2_ adsorption and release at lower, intermediate temperatures. To further assess the relevance of the surface activation during the reducing pretreatment and the concomitant formation of Ni-BaO interface, Figure 4b reports the catalytic activity of NiBaZr­(AS1.1) reduced at 350 °C (R 350, where the sample surface is only partially reduced) and at 550 °C (R 550, achieving instead the full reduction of the surface and the formation of the active interface). R 350 reached lower ammonia conversion values, with a lower reaction rate (0.73 μmol g^–1^ s^–1^) with respect to R 550 (1.17 μmol g^–1^ s^–1^) at 380 °C.

**Figure 4:**
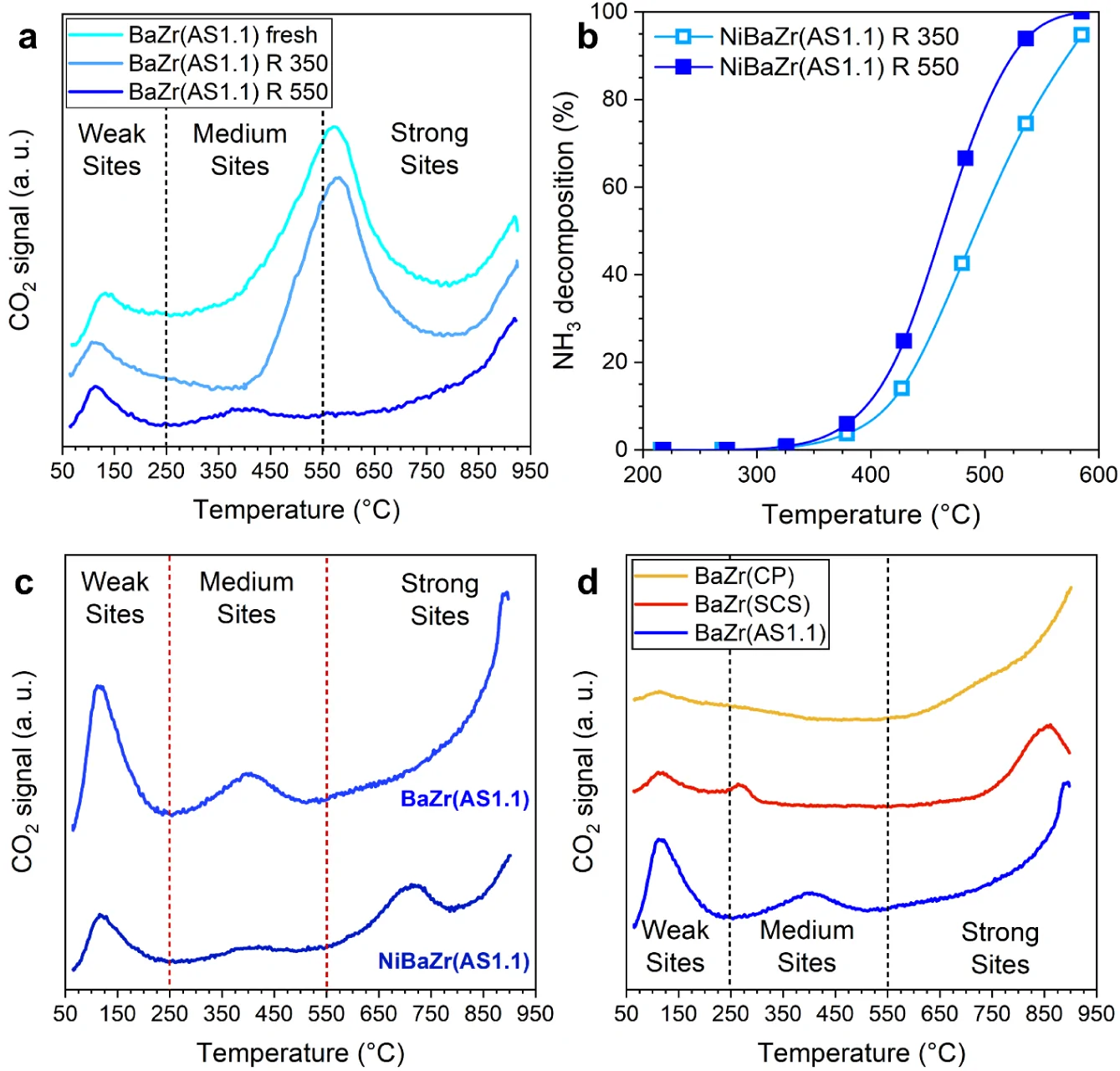
(a) CO_2_-TPD profiles of BaZr­(AS1.1) after different reduction temperatures; (b) light-off curves of NiBaZr­(AS1.1) reduced at 350 °C and 550 °C; (c) comparison of CO_2_-TPD profiles for BaZr­(AS1.1) support and NiBaZr­(AS1.1) catalyst; (d) CO_2_-TPD of BaZr­(CP), BaZr­(SCS), and BaZr­(AS1.1) supports.

Figure 4c compares CO_2_-TPD profiles of BaZr­(AS1.1) before and after Ni deposition. The overall intensity of CO_2_ desorption decreases after metal addition because of the presence of Ni particles which occupy available surface basic sites, consistent with prior observations.^48^ Interestingly, a new high-temperature peak appears at 714 °C on NiBaZr­(AS1.1). Since Ni surface cannot chemisorb CO_2_, this peak could likely be associated with strongly bonded CO_2_ at the interface between Ni nanoparticles and BaO sites, where high electron density enhances CO_2_ adsorption. These results further confirm the strong metal-support interaction, possibly indicating the formation of a Ni-BaO interface, which provides highly active basic sites relevant to catalytic activity.

TPD profiles (Figure 4d) of BaZr­(AS1.1), BaZr­(SCS), and BaZr­(CP) after H_2_ pretreatment at 550 °C reveal multiple CO_2_ desorption features related to physisorbed and chemisorbed species. The initial low-temperature desorption correlates with SSA: the larger the surface, the greater the number of available weak basic sites for physisorbed CO_2_. In the medium basicity region, broad peaks are observed only for BaZr­(AS1.1) and BaZr­(SCS), with maxima at 402 °C and 264 °C, respectively. These are reasonably generated from the decomposition of surface BaCO_3_ during reduction and the concurrent formation of active basic sites. Among all samples, BaZr­(AS1.1) exhibits the highest surface basicity, indicating that a higher amount of BaCO_3_ can serve as a precursor for basic site generation when properly activated, and again explaining the higher catalytic activity of this material. These results underscore the beneficial interplay between NiO reduction and BaO formation during H_2_ pretreatment, generating Ni-BaO entities: the catalyst, retaining surface carbonates subsequently decomposed during reduction at a suitable temperature, exhibits improved activity due to the formation of BaO basic sites interacting with Ni nanoparticles, as inferred from TPR and CO_2_-TPD data.

To further corroborate the formation of BaO sites, a detailed investigation on BaZr­(AS1.1) was carried out using a multi-technique *in situ* approach (XRD, Raman, DRIFTS), simulating the CO_2_-TPD experiment. Key findings are summarized in Figure 5. The reduction process was monitored via XRD (Figure 5a) and Raman spectroscopy (Figure 5b), both showing a decrease in BaCO_3_-related signals with respect to the perovskite lattice peaks. This confirms its partial decomposition under reducing conditions with the formation of BaO. Subsequent CO_2_ exposure at 60 °C, analyzed by *in situ* XRD (Figure 5c), revealed no significant structural changes, suggesting that CO_2_ adsorption occurs mainly at the surface level. Indeed, DRIFT spectroscopy (Figure 5d) identified the formation of various carbonate species, attributed to CO_2_ adsorption on surface BaO sites, with bands at 1630 cm^–1^ (COO symmetric and asymmetric stretching of unidentate complexes),^49,50^ 1573 cm^–1^ (COO asymmetric stretching of bridging complexes),^49^ 1308 cm^–1^ (asymmetric stretching of bidentate complexes),^51^ and 1056 cm^–1^ (CO_3_^2–^ symmetric stretching vibration).^35,52^ These signals persisted even after prolonged exposure under inert atmosphere (Figure 5d), indicating stable chemisorbed species. Upon heating to 450 °C (Figure 5e), a progressive decrease in these bands was observed, aligning with CO_2_-TPD results and confirming desorption from weak and medium basic sites. The results are in good agreement with what was observed in the conventional CO_2_-TPD experiment, and confirm that CO_2_ adsorption on BaZr­(AS1.1) primarily involves weak and medium basic BaO sites formed from surface BaCO_3_ decomposition.

**Figure 5:**
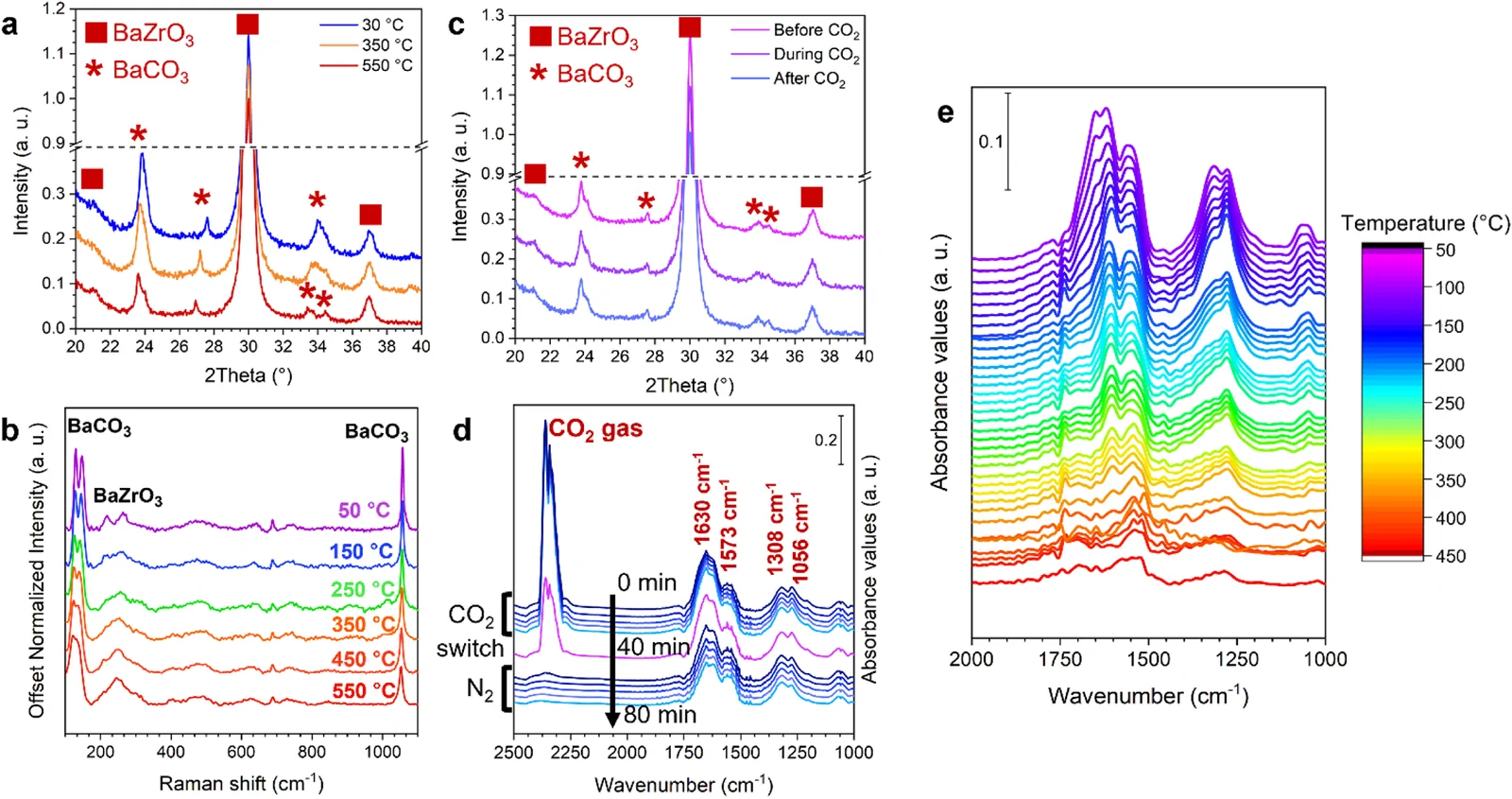
(a) *In situ* XRD and (b) *in situ* Raman of BaZr­(AS1.1) treated at 550 °C in 5% H_2_; (c) *in situ* XRD and (d) *in situ* DRIFTS of reduced BaZr­(AS1.1) during CO_2_ exposure at 60 °C; (e) *in situ* DRIFTS of BaZr­(AS1.1) during the heating ramp up to 450 °C.

The correlation between these findings and data from CO_2_-TPD and H_2_-TPR supports the hypothesis of BaO surface formation in interaction with Ni, which plays a key role in enhancing the catalytic activity for ammonia decomposition on these materials.

In conclusion, these results highlight the beneficial role of BaCO_3_ that can be exploited by suitable H_2_ reduction to generate BaO basic sites interacting with Ni nanoparticles, leading to highly active catalysts, as evidenced by TPR and CO_2_-TPD analyses.

### 2.4 Ni-BaZrO_3_ Ammonia Adsorption and Decomposition Mechanism

To get insights also on ammonia adsorption on the different materials and to further investigate the underlying mechanism(s) behind the observed differences in catalytic activity for NH_3_ decomposition, TPSR experiments were carried out on the three BaZrO_3_-supported catalysts and the three internal benchmarks following a previously established protocol,^53^ with results shown in Figure 6. Prior to the TPSR test, catalytic tests were performed at 480 °C (results reported in Figure S14) at a reduced space velocity to enhance NH_3_ adsorption and the formation of adsorbed intermediates (NH_2_^ad^, NH^ad^, H^ad^, and N^ad^). During the desorption ramp in He, NH_3_, H_2_, and N_2_ signals were monitored via mass spectrometry to track recombination and desorption of surface species.

**Figure 6:**
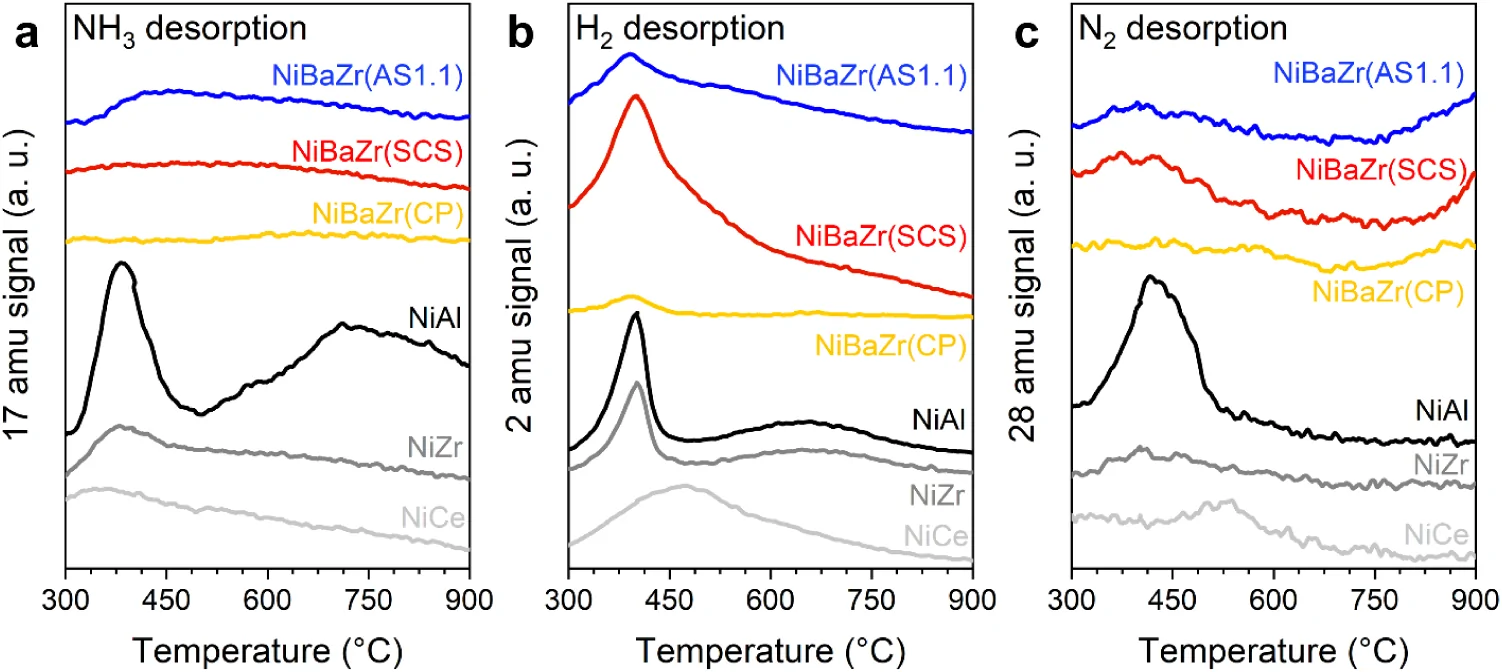
(a) NH_3_ signal, (b) H_2_ signal, and (c) N_2_ signal for Ni-based catalysts during the desorption part of TPSR.

Figure 6a shows the NH_3_ desorption profiles for the tested catalysts. NiBaZr­(AS1.1) exhibits delayed NH_3_ desorption compared to NiAl, NiZr, and NiCe, while NiBaZr­(SCS) and NiBaZr­(CP) show minimal NH_3_ release. Delayed and/or limited NH_3_ desorption is desirable, as weakly adsorbed ammonia tends to desorb unreacted or recombine into NH_2_^ad^/NH^ad^ species rather than dehydrogenating.^53,54^ Among the catalysts, Ni-BaZrO_3_ samples show minimal NH_3_ release, whereas NiCe and NiZr rapidly desorb ammonia, and NiAl displays the highest NH_3_ release. Compared to the other NiBaZr catalysts, NiBaZr­(AS1.1), which also has a higher specific surface area, is more effective in adsorbing and cracking ammonia. From the NH_3_ desorption results, we can thus qualitatively assume that the population of surface acid sites, which are responsible for NH_3_ adsorption, follows the trend NiAl ≫ NiZr > NiCe ≈ NiBaZr­(AS1.1) > NiBaZr­(SCS) ≈ NiBaZr­(CP). Therefore, a suitable balance between strongly bound NH_3_^ad^/NH_2_^ad^/NH^ad^ species and an adequate number of accessible acid sites could explain the better catalytic activity of the NiBaZr­(AS1.1) catalyst among the other counterparts. These results also suggest that NH_2_^ad^ and NH^ad^ species are more strongly bound to BaZrO_3_ surfaces and tend to decompose to H^ad^ and N^ad^ rather than recombine into NH_3_.^53^ Figure 6b,c show H_2_ and N_2_ desorption profiles, respectively. In contrast to NH_3_, efficient ammonia decomposition correlates with low-temperature H_2_ and N_2_ desorption, allowing faster regeneration of active sites.^55^ Ni-BaZrO_3_ catalysts display a single H_2_ desorption peak around 400 °C, indicating fast desorption. In contrast, NiZr and NiAl exhibit a two-step H_2_ release (peaks at 400 °C and 650 °C), suggesting stronger H_2_ binding on some active sites. NiCe shows a single peak, shifted to 480 °C. N_2_ desorption (Figure 6c) also strongly correlates with catalytic activity: NiBaZr­(AS1.1) and NiBaZr­(SCS) release N_2_ earlier than the other samples, with NiAl following closely. This behavior highlights that, beyond surface basicity, the high activity of Ni-BaZrO_3_ catalysts also stems from a proper amount of surface acid sites strongly anchoring NH_3_, effective NH_3_ activation, and moderate Ni–N and Ni–H interactions, enabling faster desorption of reaction products. This can be traced back to the formation of Ni-BaO in a strong interaction, with BaO sites acting as electron donors to Ni nanoparticles, facilitating the desorption of reaction products.

Considering the constant evolution of the catalyst surface under reaction conditions, to better understand the NH_3_ decomposition mechanism on Ni-BaZrO_3_ catalysts, NiBaZr­(AS1.1) and NiBaZr­(CP) were also studied by *in situ* DRIFTS experiments. These two samples were selected for the analysis due to their clearly different reactivity to understand the role of crystallinity, SSA, defectivity, surface acid/base properties, and, most notably, BaCO_3_ content. Additionally, NiBaZr­(CP) catalytic activity closely resembles the behavior of the other benchmark catalysts (NiZr and NiAl, specifically). Figure 7 shows IR spectra from 100 °C to 480 °C in different spectral ranges while exposing the sample to 2.5% NH_3_. Full spectra are instead reported in Figure S15. In Figure 7a,b, gaseous NH_3_ bands (933 and 969 cm^–1^) and NH_3_ adsorbed on Lewis acid sites (1000–1200 cm^–1^)^56^ decrease with temperature due to the catalytic decomposition, then increase again at 400 °C with the appearance of a 1404 cm^–1^ band, indicating NH_4_^+^ formation on Brønsted acid sites;^57^ this is more evident on the CP sample. This behavior is probably due to the activation of new adsorption sites on the catalyst surface at high temperature, which is further evidence of catalyst evolution under reaction atmosphere. A broad band at 1600–1500 cm^–1^, assigned to NH_2_ species from NH_3_ deprotonation, is instead more intense on NiBaZr­(AS1.1), indicating that on this sample, NH_3_ activation and dehydrogenation are faster.^56,57^ At low temperatures (100–150 °C), the bands associated with species adsorbed on Lewis acid sites are more intense for NiBaZr­(AS1.1), indicating a larger number of accessible surface acid sites that help NH_3_ adsorption on the catalyst surface, in good agreement with the NH_3_-TPSR results. This difference is more evident in the 4000–3000 cm^–1^ range (Figure 7c,d), where bands at ∼3500 cm^–1^ (adsorbed NH_3_ on Lewis acid sites) and ∼3200 cm^–1^ (NH_4_^+^ adsorbed on Brønsted acid sites) are observed.^56,57^ These bands are clearly visible only on NiBaZr­(AS1.1), further supporting the previous assumptions. Notably, NiBaZr­(AS1.1) shows also stronger −OH vibrations (3800–3600 cm^–1^), suggesting greater H atom mobility, which may promote H_2_ recombination on the support as well as on Ni particles. Indeed, H_2_ spillover during NH_3_ decomposition has been demonstrated to be beneficial for Ba-based perovskites reactivity, suggesting an active role of the perovskite structure in the NH_3_ decomposition mechanism.^11,15^ This assumption can be somehow confirmed by the appearance from 350 °C of unconventional peaks at 2154 cm^–1^ and 1990 cm^–1^ (Figure 7e,f), which can be attributed to N–N species adsorbed on the metal-support interface.^10,58^ Particularly, Ogasawara et al. hypothesized that the N_2_ formation reaction preferentially occurs at the interface between Ni and BaTiO_3–*x*_-N_*y*_, with N–N species probably adsorbed on Ti cations due to the appearance of these two bands.^10,59^ Therefore, the N_2_ formation reaction could occur at defect sites at the interface, which are abundant on NiBaZr­(AS1.1) as shown by Raman spectroscopy. Dinitrogen bands are much more intense on the NiBaZr­(AS1.1) sample, indicating a higher/faster formation of N–N bonds on this catalyst. This is a further explanation of the superior activity of NiBaZr­(AS1.1) in comparison with NiBaZr­(CP). It should be mentioned, however, that the attribution of these bands related to N–N species is still debated^60^ since, to the best of our knowledge, in the literature specific studies are currently scarce, if not absent.

**Figure 7:**
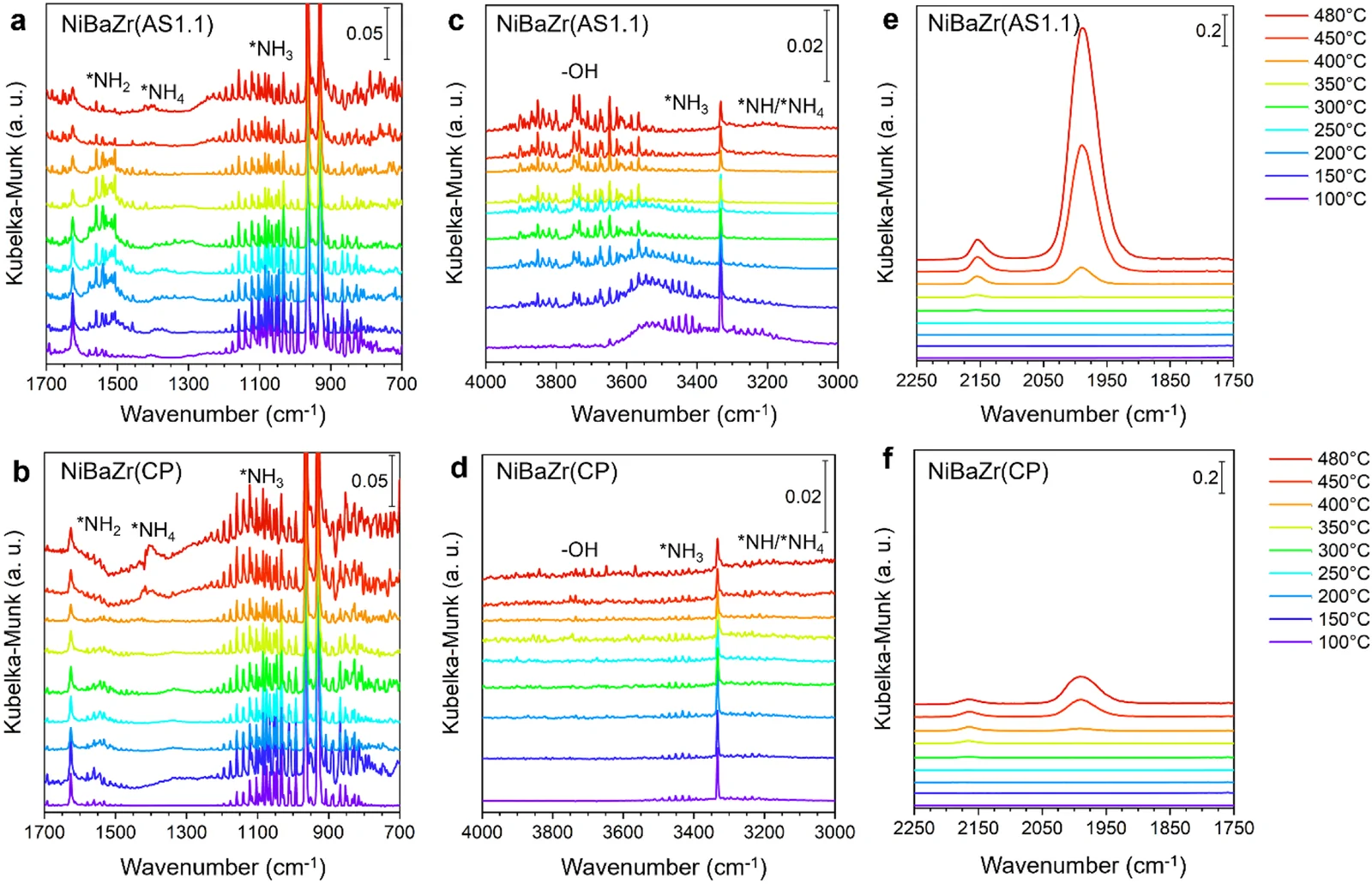
Different magnifications of DRIFT spectra during NH_3_ decomposition reaction using NiBaZr­(AS1.1) and NiBaZr­(CP) as catalysts, respectively: (a, b) 1700–700 cm^–1^ region; (c, d) 4000–3000 cm^–1^ region; (e, f) 2250–1750 cm^–1^ region.

Additional DRIFT spectra were collected to understand the catalysts’ ability to adsorb ammonia and release adsorbed species. Samples were exposed to NH_3_ at 450 °C, then purged with pure N_2_ for 15 min. Figure 8 shows the evolution of the dinitrogen species region for NiBaZr­(AS1.1) (Figure 8a) and NiBaZr­(CP) (Figure 8b). After the gas switch, the bands at 2154 cm^–1^ and 1990 cm^–1^ significantly decrease for NiBaZr­(AS1.1), while remaining nearly unchanged for NiBaZr­(CP). This suggests that adsorbed species desorb more readily from NiBaZr­(AS1.1), likely due to enhanced electron transfer at the Ni-BaZrO_3_ interface, facilitated by BaO species donating electrons to Ni. Overall, these results support the conclusion that H and/or N recombination and desorption preferentially occur at the more electronically active Ni-BaZr­(AS1.1) interface.

**Figure 8:**
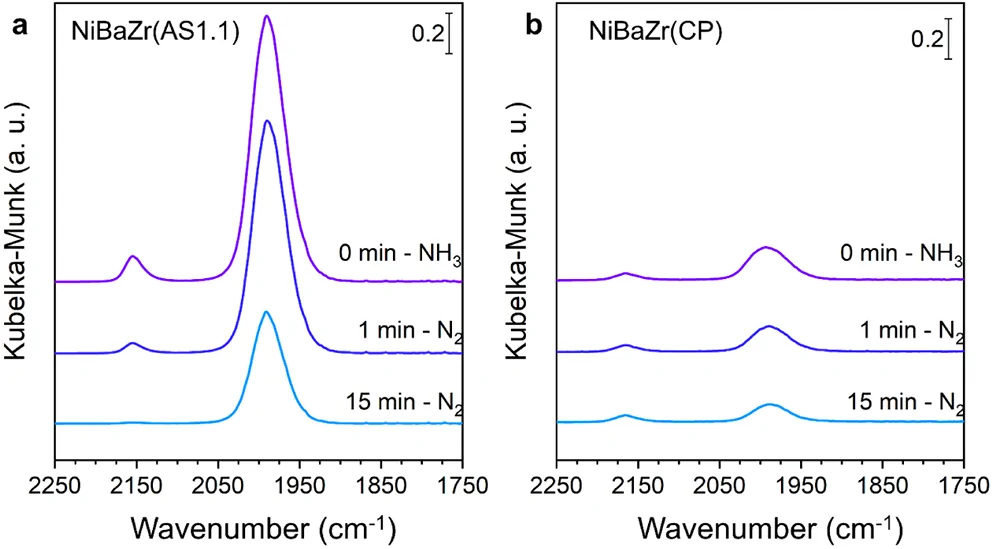
Magnification of the 2250–1750 cm^–1^ region of DRIFT spectra during NH_3_ decomposition reaction and consecutive N_2_ purge at 450 °C: (a) NiBaZr­(AS1.1); (b) NiBaZr­(CP).

## 3 Conclusions

In this work, barium zirconate was successfully synthesized using a modified sol–gel method with acetate precursors, combined with a mechanochemical approach for Ni deposition. By adjusting the citric acid-to-cation ratio (CA/C^+^), the synthesis enables control over SSA, porosity, and BaCO_3_ segregation. Careful analysis of BaCO_3_ decomposition, obtained by combining different characterization techniques, revealed the role of carbonates on the formation of active Ni-BaO entities. From TPR analysis, two distinct types of carbonates could be observed: surface and bulk species. Upon reduction at a suitable temperature, surface BaCO_3_ decomposition was shown to form BaO, generating new basic sites strongly interacting with the active Ni phase. CO_2_-TPD results confirm this interaction, which contributes to improved metal-support interplay. TPSR experiments allow to understand that Ni-BaZrO_3_ catalysts strongly interact with NH_3_ while efficiently releasing N_2_ and H_2_, promoting fast NH_3_ dehydrogenation and product desorption. DRIFTS analysis reveals the formation of adsorbed dinitrogen species and faster desorption from the surface of NiBaZr­(AS1.1) compared to NiBaZr­(CP). Overall, the results highlight the enhanced surface defectivity, reducibility, and basicity of the sample prepared by acetate synthesis leading to its superior catalytic activity with double reaction rate with respect to nickel supported on conventionally prepared BaZrO_3_ and on common oxide supports (CeO_2_, Al_2_O_3_, ZrO_2_).

Notably, the acetate synthesis combined with the mechanochemical Ni deposition significantly reduces the environmental impact by minimizing NO_*x*_ emissions, solvent use, and water waste. Compared to conventional methods, the acetate route yields perovskites with higher SSA and improved porosity, and dry milling can be exploited for metal deposition without altering the textural properties. These findings suggest that controlling BaCO_3_ segregation and subsequent decomposition could be key to forming active basic sites efficiently interacting with the metal phase, offering a promising strategy for developing efficient Ru-free ammonia decomposition catalysts with minimal environmental impact.

## 4 Methods

### 4.1 Synthesis of Ni-BaZrO_3_ Catalysts

All chemicals used in the syntheses—Ba­(NO_3_)_2_, ZrO­(NO_3_)_2_, Ba­(CH_3_COO)_2_, Zr­(CH_3_COO)_4_, and citric acid (C_6_H_8_O_7_)—were supplied by Treibacher Industrie AG (Austria). Scheme 1 in the Supporting Information illustrates the three synthesis routes employed for the preparation of BaZrO_3_ supports, with each method yielding 30 g of perovskite.

The Acetate Synthesis (AS) was developed as a modification of the conventional citrate sol–gel method in collaboration with Treibacher Industrie AG. Appropriate amounts of barium and zirconium acetates were dissolved in 450 mL of deionized water and stirred at room temperature for 30 min. A citric acid (CA) solution was then added, with the CA-to-metal cation molar ratio (CA/C^+^) varied between 0.4 and 2.0 to assess its impact on the final oxide morphology. Ammonia (24%) was added dropwise until the solution reached pH 7. The mixture was then heated to 80 °C to evaporate water, forming a wet gel, which was subsequently transferred to a muffle furnace and treated at 220 °C for 12 h. This resulted in a dry brown sponge (Figure S16), which was ground to obtain the final product.

Conventional Self-Combustion Synthesis (SCS) was carried out starting with Ba and Zr nitrates, following a literature recipe.^8^ Precursors were dissolved and stirred for 1 h in 450 mL of water at 60 °C; then, CA solution was added. A CA/C^+^ ratio of 1.1 was chosen. The solution was then heated at 80 °C until a dried gel was formed. The gel was then heated at 350 °C to guarantee a total combustion of the organic network.

The coprecipitation (CP) route was also reproduced from the literature.^22^ Barium and Zirconium nitrates were dissolved in 450 mL of water and stirred for 60 min. 800 mL of NaOH solution (12.5 M) was heated to 80 °C in the meanwhile. The metal solution was then added to NaOH with a constant rate of 220 mL min^–1^. Precipitates formed immediately after the addition. The solution was then stirred at 80 °C for 30 min, and then centrifuged and washed 3 times. After washing, the material was dried overnight at 100 °C and then manually ground.

All the materials were calcined at 800 °C for 6 h in static air separately. The powders obtained were labeled BaZrO_3_ AS (Acetate Synthesis), SCS (Self-Combustion Synthesis), and CP (Coprecipitation). Since the effect of the citric acid concentration was studied, BaZrO_3_ AS samples were labeled with a number indicating the CA/C^+^ ratio.

Ni-BaZrO_3_ catalysts (10 wt % Ni) were obtained by mechanochemical synthesis with a Pulverisette 23 Minimill (Fritsch) using mild-energy conditions. Ni acetate (Sigma-Aldrich, 99.9%) was used as precursor. BaZrO_3_ support (900 mg) and Ni acetate (424.1 mg) were preliminarily mixed and then put inside a 15 mL ZrO_2_ jar with a 15 mm sphere. Milling was carried out at 15 Hz for 20 min. The ball-to-powder weight ratio was fixed at 10. Milled samples were then calcined at 550 °C for 2 h in static air.

CeO_2_ (Rhodia), Al_2_O_3_ (Sasol), and ZrO_2_ (Treibacher) were used to prepare reference materials. Ni acetate was deposited as described above. Samples were then calcined at 550 °C for 2 h in static air.

Additional details on the synthesis procedure, together with the corresponding chemical reactions occurring during AS, are outlined in the SI, together with a brief discussion of the environmental impact of the proposed synthesis approaches.

### 4.2 Characterization of BaZrO_3_ Supports and Ni-BaZrO_3_ Catalysts

Specific surface area and pore size measurements were carried out by nitrogen adsorption/desorption at −196 °C in a Micromeritics TriStar 3000 apparatus. Elemental composition was analyzed with a Malvern Panalytical Zetium XRF spectrometer. XRD analyses were done with a Philips PW3040/60 X’Pert PRO diffraction system using Cu Kα radiation. TGA was performed using a TGA 5500 (Waters TA Instruments). A FEG-SEM (JEOL JSM-7600F) equipped with energy-dispersive X-ray spectroscopy (EDS, Oxford X-Max) was used for the acquisition of micrographies, using secondary electrons, and elemental maps. A FTIR spectrometer (Thermo Scientific Nicolet iS50 FTIR) was used for ATR measurements and for *in situ* DRIFTS of CO_2_-TPD and NH_3_ decomposition. An Xplora Plus Micro-Raman (Horiba) spectrometer was used for Raman spectra collection. TPR and CO_2_-TPD were carried out in a Micromeritics AutoChem II 2920 analyzer equipped with a TCD detector and connected to an OmniStar GSD 350 mass spectrometer (Pfeiffer Vacuum). A more detailed description of all the characterization techniques is reported in the SI.

### 4.3 Ammonia Decomposition Activity of Ni-BaZrO_3_ Catalysts

Ammonia decomposition reaction tests were carried out on 50 mg of sample loaded on a quartz wool bed in a tubular quartz reactor (ID 6 mm) placed in a vertical tubular furnace. Samples were pretreated in 5% of H_2_ in He (40 mL min^–1^) at 550 °C for 1 h and then cooled down in pure He. Catalytic tests were carried out by feeding 5.0% of NH_3_ in He (30 mL min^–1^, with a GHSV ≈ 30,000 h^–1^). He, NH_3_, N_2_, and H_2_ were monitored by a GSD 350 Omnistar mass spectrometer (Pfeiffer Vacuum). Isotherms of 15 min were maintained every 50 °C starting from 200 °C to 550 °C. NH_3_ conversion at a defined *T* was calculated as follows:

3 $${\mathrm{\%NH}}_{3}\left(T\right)=\frac{{[{\mathrm{NH}}_{3}]}_{\mathrm{in}}-{[{\mathrm{NH}}_{3}]}_{T}}{{[{\mathrm{NH}}_{3}]}_{\mathrm{in}}}\cdot 100$$

where ${[{\mathrm{NH}}_{3}]}_{\mathrm{in}}$ and ${[{\mathrm{NH}}_{3}]}_{T}$ are the initial percentage concentration and the average percentage concentration at isotherm *T*, respectively.

Reaction rates were measured at the isothermal step at 380 °C, in order to ensure the kinetic regime, i.e., conversion values below 10%, for each sample. The reaction rate *r*_cat_ (reaction rate per gram of catalyst) was then calculated using the following equation:

4 $$r_{\mathrm{cat}}=\frac{\%X\left(380^{{}^{\circ}}C\right)\cdot F}{m_{\mathrm{cat}}}{[\mathrm{mol g}}^{-1}{\;s}^{-1}]$$

where %*X*(380 °C) is the ammonia conversion measured at 380 °C, *F* is the ammonia molar flow rate [mol s^–1^], and *m*_cat_ is the mass of catalyst [g].

## Supplementary Material



## Notes

Details on characterization methods (XRF, BET/BJH, XRD, TGA, ATR, Raman, SEM/EDS, H_2_-TPR, CO_2_-TPD, DRIFTS, TPSR); N_2_ isotherms of the most representative BaZrO_3_ samples (Figure S1) and resulting pore size distribution (Figure S2); pore distribution profiles of BaZr­(AS1.1) and NiBaZr­(AS1.1) (Figure S3); X-ray diffractograms of BaZr­(AS0.8), (AS1.1), (AS1.8), and (AS2.0) (Figures S4 and S5) and NiBaZr­(CP), NiBaZr­(SCS), and NiBaZr­(AS1.1) (Figure S6); TG analysis of Ni acetate and Ni acetate deposited on BaZr­(AS1.1), BaZr­(SCS), and BaZr­(CP) (Figure S7) with data discussion; Raman spectra of the most representative samples (Figure S8) with peak attribution (Table S1) and data discussion; comparison between NiBaZr­(AS 1.1) performance for ammonia decomposition with benchmark materials from the literature (Table S2) and with internal milled catalysts (Table S3); light-off curves (Figure S9) and H_2_ production at 480 °C (Figure S10) of the studied Ni-based catalysts; X-ray diffractograms of Ni-BaZrO_3_ catalysts after NH_3_ decomposition test (Figure S11) and after Time-on-Stream test (Figure S12); TPR analysis of Ni-based catalysts (Figure S13) with quantitative analysis of gaseous species consumed and produced during reduction of BaZr­(AS1.1) and NiBaZr­(AS1.1) (Table S4); ammonia decomposition activity at 480 °C (Figure S14) before TPSR characterization; full spectra collected during the DRIFTS analysis of ammonia decomposition reactivity of NiBaZr­(AS1.1) and NiBaZr­(CP) (Figure S15); schematized synthesis methods (Scheme 1) with considerations on acetate synthesis and its environmental impact (Figure S16) (PDF)

## References

[1] Ma N., Zhao W., Wang W., Li X., Zhou H. (2024). Large Scale
of Green Hydrogen Storage: Opportunities and Challenges. Int. J. Hydrogen Energy.

[2] Ishaq H., Crawford C. (2024). Review and Evaluation
of Sustainable Ammonia Production,
Storage and Utilization. Energy Convers. Manage.

[3] Mekonnin A. S., Wacławiak K., Humayun M., Zhang S., Ullah H. (2025). Hydrogen Storage
Technology, and Its Challenges: A Review. Catalysts.

[4] Han T., Wei L., Xie S., Liu Y., Dai H., Deng J. (2025). Catalyst Design
for Ammonia Decomposition: An Overview. Ind.
Chem. Mater.

[5] Zhu N., Yang F., Hong Y., Liang J. (2025). Hydrogen Production
from Ammonia Decomposition: Advances in Ru- and Ni-Based Catalysts. Int. J. Hydrogen Energy.

[6] Farooqi A. S., Ramli R. M., Hasnain S. M. W. U., Farooqi A. S., Sharaf
Addin A. H., Abdullah B., Arslan M. T., Farooqi S. A. (2025). A Comprehensive
Review on Hydrogen Production via Catalytic Ammonia Decomposition
over Ni-Based Catalysts. Int. J. Hydrogen Energy.

[7] Solanki B. S., Lim H., Yoon S. J., Ham H. C., Park H. S., Lee H. E., Lee S. H. (2025). Recent
Advancement of Non-Noble Metal Catalysts for
Hydrogen Production by NH_3_ Decomposition. Renewable Sustainable Energy Rev.

[8] Okura K., Miyazaki K., Muroyama H., Matsui T., Eguchi K. (2018). Ammonia Decomposition
over Ni Catalysts Supported on Perovskite-Type Oxides for the on-Site
Generation of Hydrogen. RSC Adv.

[9] Pinzón M., Sánchez-Sánchez A., Romero A., de la Osa A. R., Sánchez P. (2022). Self-Combustion
Ni and Co-Based Perovskites as Catalyst
Precursors for Ammonia Decomposition. Effect of Ce and Mg Doping. Fuel.

[10] Ogasawara K., Miyazaki M., Miyashita K., Abe H., Niwa Y., Sasase M., Kitano M., Hosono H. (2023). Ammonia Decomposition
over Water-Durable Hexagonal BaTiO_3–x_N_y_-Supported Ni Catalysts. Adv. Energy Mater.

[11] Chen Z., Jiang B., Lu Z., Huang J., Shi J., Liu M. (2025). Strong Metal-Support
Interaction-Induced Hydrogen Spillover Boosts
Thermocatalytic Ammonia Decomposition. Small.

[12] Martín C., Sánchez P., de Lucas-Consuegra A., Romero J., Pinzón M., de la Osa A. R. (2026). The Potential of Glycerine as a Fuel in the Synthesis
of Highly Active Nickel-Based Perovskites for Ammonia Decomposition. J. Environ. Chem. Eng.

[13] Wang Z., Qu Y., Shen X., Cai Z. (2019). Ruthenium
Catalyst Supported on Ba
Modified ZrO_2_ for Ammonia Decomposition to CO_x_-Free Hydrogen. Int. J. Hydrogen Energy.

[14] Qin C., Ruan S., He C., Zhang L. (2024). Nickel Perovskite Catalysts
for Ammonia Decomposition: DFT Calculations and Microreaction Kinetics. Colloids Surf., A.

[15] Wang Y., Mao X., Yang J., Wang J., Guan W., Chen J., Han B., Tian Z. (2022). One-Step Synthesis of Ni/Yttrium-Doped Barium Zirconates
Catalyst for on-Site Hydrogen Production from NH_3_ Decomposition. Int. J. Hydrogen Energy.

[16] Ren H., Zhao Y., Luo Z., Hu E., Yin G., Huang Z. (2026). Experimental and Theoretical Study
of BaZrO_3_ Supported
Ru-Ni Catalysts for Ammonia Decomposition Reaction. Fuel.

[17] Regalado
Vera C. Y., Ding H., Peterson D., Gibbons W. T., Zhou M., Ding D. (2021). A Mini-Review on Proton Conduction
of BaZrO_3_-Based Perovskite Electrolytes. JPhys. Energy.

[18] Miyazaki K., Okanishi T., Muroyama H., Matsui T., Eguchi K. (2017). Development
of Ni–Ba­(Zr,Y)­O_3_ Cermet Anodes for Direct Ammonia-Fueled
Solid Oxide Fuel Cells. J. Power Sources.

[19] Bello I. T., Karki S., Zheng S., Ruan J., Kumari A., Geng Y., Patel S., Li Z., Kazempoor P., Bian W., Ling Y., Ghamarian I., Liu Y., Wang B., Shao Z., Ni M., Ding H. (2025). Multiscale
Engineering of BaZr_1‑X_Y_x_O_3‑δ_ -Based Protonic Ceramics: A Critical Review of Defect Chemistry,
Interface Design, and Computational Insights. Energy Rev.

[20] Ya Y., Xu Y. S., Elbanna A. M., Liu Y., Sun B., Cheng X. (2025). Review of
Direct Ammonia Solid Oxide Fuel Cells: Low Temperature
Cell Structure and Ammonia Decomposition Strategies. Renewable Sustainable Energy Rev.

[21] Manju P., Ajith M. R., Jaiswal-Nagar D. (2019). Synthesis
and Characterization of
BaZrO_3_ Nanoparticles by Citrate-Nitrate Sol-Gel Auto-Combustion
Technique: Systematic Study for the Formation of Dense BaZrO_3_ Ceramics. J. Eur. Ceram. Soc.

[22] Boschini F., Rulmont A., Cloots R., Vertruyen B. (2009). Rapid Synthesis
of Submicron Crystalline Barium Zirconate BaZrO_3_ by Precipitation
in Aqueous Basic Solution below 100 °C. J. Eur. Ceram. Soc.

[23] Babu A., Tirumalarao D., Das S., Dixit V., Sruthy S. P., Vijayan V., Jaiswal-Nagar D. (2022). Effect of
PH Variation on Citrate
Nitrate Sol-Gels Obtained from Auto-Combustion Method: Synthesis,
Calculations and Characterisations of Extremely Dense BaZrO_3_ Ceramic. Open Ceram.

[24] Dai H., Kou H., Wang H., Bi L. (2018). Electrochemical Performance
of Protonic
Ceramic Fuel Cells with Stable BaZrO_3_-Based Electrolyte:
A Mini-Review. Electrochem. Commun.

[25] Manju P., Babu A., Sruthy S. P., Tirumalarao D., Abhijith P. V., Kamble V. B., Jaiswal-Nagar D. (2022). Variation
of Citrate to Metal Cation Ratio for Dense and Phase Pure BaZrO_3_ via Autocombustion Synthesis. J. Am.
Ceram. Soc.

[26] Xiang X., Jing J., Wang H., Wang X., Li M., Wang F., Chen J., Guo Y., He L., Zhao H., Yang Z. (2025). Synergistic A-Site
Compensation and
Oxygen Vacancy Engineering Boost High-Entropy Electrolyte Performance
in Protonic Ceramic Fuel Cells. Nano Lett.

[27] Zhang W., Li D., Liu C., Jiang Z., Gao C., Shen L., Liu Q., Qiu R., Gu H., Lian C., Xu J., Zhu M. (2024). Facilitated CO_2_ Hydrogenation by Strong Metal-Support
Interaction between Ni and BaCO_3_. Chem. Catal.

[28] Braga A., Felli A., Strazzolini A., Danielis M., Colussi S., Trovarelli A. (2025). Tuning A-Site Chemistry in AZrO_3_ Perovskites
for Integrated CO_2_ Capture and Methanation. Appl. Catal. A Gen.

[29] Sato K., Nagaoka K., Miyahara S. I., Tsujimaru K., Wada Y., Toriyama T., Yamamoto T., Matsumura S., Inazu K., Mohri H., Iwasa T., Taketsugu T. (2021). Barium Oxide
Encapsulating Cobalt Nanoparticles Supported on Magnesium Oxide: Active
Non-Noble Metal Catalysts for Ammonia Synthesis under Mild Reaction
Conditions. ACS Catal.

[30] Hayashi S., Takeuchi Y., Naito T., De Silva K. K. H., Sato K., Toriyama T., Yamamoto T., Murakami Y., Nagaoka K. (2026). Design of
Alkaline Earth-Doped Co/MgO Catalysts for Ammonia Decomposition. ChemSuschem.

[31] Royer S., Duprez D., Can F., Courtois X., Batiot-Dupeyrat C., Laassiri S., Alamdari H. (2014). Perovskites as Substitutes of Noble
Metals for Heterogeneous Catalysis: Dream or Reality. Chem. Rev.

[32] Danielis M., Felli A., Zampol M., Fonda N., Brüner P., Grehl T., Furlani E., Maschio S., Colussi S., Trovarelli A. (2024). Tuning Chemical
and Morphological Properties of Ceria
Nanopowders by Mechanochemistry. ACS Omega.

[33] Parida S., Rout S. K., Cavalcante L. S., Sinha E., Li M. S., Subramanian V., Gupta N., Gupta V. R., Varela J. A., Longo E. (2012). Structural Refinement, Optical and Microwave Dielectric Properties
of BaZrO_3_. Ceram. Int.

[34] Yan H., Zhang D., Xu J., Lu Y., Liu Y., Qiu K., Zhang Y., Luo Y. (2014). Solution Growth of NiO Nanosheets
Supported on Ni Foam as High-Performance Electrodes for Supercapacitors. Nanoscale Res. Lett.

[35] Pasierb P., Komornicki S., Rokita M., Rȩkas M. (2001). Structural
Properties of Li_2_CO_3_–BaCO_3_ System Derived from IR and Raman Spectroscopy. J. Mol. Struct.

[36] Toulouse C., Amoroso D., Xin C., Veber P., Hatnean M. C., Balakrishnan G., Maglione M., Ghosez P., Kreisel J., Guennou M. (2019). Lattice Dynamics and Raman Spectrum
of BaZrO_3_ Single Crystals. Phys.
Rev. B.

[37] Shi F., Dong H., Liu Q., Yang J., Ren S., Sun H., Xiong J. (2017). Investigation
and Theoretical Calculation of the Lattice
Vibrational Spectra of BaZrO_3_ Ceramic. J. Mater. Sci.: Mater. Electron.

[38] Zhang H., Alhamed Y. A., Kojima Y., Al-Zahrani A. A., Miyaoka H., Petrov L. A. (2014). Structure and Catalytic
Properties
of Ni/MWCNTs and Ni/AC Catalysts for Hydrogen Production via Ammonia
Decomposition. Int. J. Hydrogen Energy.

[39] Li Y., Wen J., Ali A. M., Duan M., Zhu W., Zhang H., Chen C., Li Y. (2018). Size Structure–Catalytic Performance
Correlation of Supported Ni/MCF-17 Catalysts for CO_x_-Free
Hydrogen Production. Chem. Commun.

[40] Wang Z., Liu B., Lin J. (2013). Highly Effective
Perovskite-Type BaZrO_3_ Supported
Ru Catalyst for Ammonia Synthesis. Appl. Catal.,
A.

[41] Leal
Villarroel E., Seguel J., Rodríguez P., Blanco E., Escalona N., Pecchi G., Sepúlveda C. (2023). Conversion
of Levulinic Acid over Ru/SrZrO_3_ and Ru/BaZrO_3_ Supported Basic Catalysts. ChemCatChem.

[42] Lux S., Baldauf-Sommerbauer G., Siebenhofer M. (2018). Hydrogenation of Inorganic Metal
Carbonates: A Review on Its Potential for Carbon Dioxide Utilization
and Emission Reduction. ChemSusChem.

[43] Ni Z., Djitcheu X., Gao X., Wang J., Liu H., Zhang Q. (2022). Effect of Preparation
Methods of CeO_2_ on the Properties
and Performance of Ni/CeO_2_ in CO_2_ Reforming
of CH_4_. Sci. Rep.

[44] Zhao K., Wang W., Li Z. (2016). Highly Efficient
Ni/ZrO_2_ Catalysts Prepared via Combustion Method for CO_2_ Methanation. J. CO_2_ Util.

[45] Song Y. Q., He D. H., Xu B. Q. (2008). Effects
of Preparation Methods of
ZrO_2_ Support on Catalytic Performances of Ni/ZrO_2_ Catalysts in Methane Partial Oxidation to Syngas. Appl. Catal., A.

[46] Dong W.-S., Roh H.-S., Jun K.-W., Park S.-E., Oh Y.-S. (2002). Methane
Reforming over Ni/Ce-ZrO_2_ Catalysts: Effect of Nickel Content. Appl. Catal., A.

[47] Tsuneto A., Kudo A., Saito N., Sakata T. (1992). Hydrogenation
of Solid
State Carbonates. Chem. Lett.

[48] Felli A., Danielis M., Trovarelli A., Colussi S. (2025). Parametric Study on
Ru/CeO_2_ Ammonia Decomposition Catalysts for Hydrogen Production. ChemCatChem.

[49] Durán P., Capel F., Gutierrez D., Tartaj J., Bañares M. A., Moure C. (2001). Metal Citrate Polymerized Complex Thermal Decomposition Leading to
the Synthesis of BaTiO_3_: Effects of the Precursor Structure
on the BaTiO_3_ Formation Mechanism. J. Mater. Chem.

[50] Visconti C. G., Lietti L., Tronconi E., Forzatti P., Zennaro R., Finocchio E. (2009). Fischer–Tropsch
Synthesis on a Co/Al_2_O_3_ Catalyst with CO_2_ Containing Syngas. Appl. Catal., A.

[51] Cornu D., Guesmi H., Krafft J. M., Lauron-Pernot H. (2012). Lewis Acido-Basic
Interactions between CO_2_ and MgO Surface: DFT and DRIFT
Approaches. J. Phys. Chem. C.

[52] Busca G., Buscaglia V., Leoni M., Nanni P. (1994). Solid-State and Surface
Spectroscopic Characterization of BaTiO_s_ Fine Powders. Chem. Mater.

[53] Meng S., Li S., Sun S., Bogaerts A., Liu Y., Yi Y. (2024). NH_3_ Decomposition for H2 Production by Thermal and Plasma Catalysis
Using Bimetallic Catalysts. Chem. Eng. Sci.

[54] Deng L., Lin H., Liu X., Xu J., Zhou Z., Xu M. (2021). Nickel Nanoparticles
Derived from the Direct Thermal Reduction of Ni-Containing Ca–Al
Layered Double Hydroxides for Hydrogen Generation via Ammonia Decomposition. Int. J. Hydrogen Energy.

[55] He H., Jiang H., Yang F., Liu J., Zhang W., Jin M., Li Z. (2023). Bimetallic Ni_x_Co_10‑x_/CeO_2_ as Highly Active Catalysts
to Enhance Mid-Temperature Ammonia
Decomposition: Kinetics and Synergies. Int.
J. Hydrogen Energy.

[56] Do Q. C., Kim Y., Le T. A., Kim G. J., Kim J. R., Kim T. W., Lee Y. J., Chae H. J. (2022). Facile One-Pot Synthesis of Ni-Based
Catalysts by Cation-Anion Double Hydrolysis Method as Highly Active
Ru-Free Catalysts for Green H_2_ Production via NH_3_ Decomposition. Appl. Catal., B.

[57] Ma Z., Wu X., Härelind H., Weng D., Wang B., Si Z. (2016). NH_3_-SCR
Reaction Mechanisms of NbO_x_/Ce_0.75_Zr_0.25_O_2_ Catalyst: DRIFTS and Kinetics
Studies. J. Mol. Catal. A: Chem.

[58] Ren J., Li H., Lou H., Zhou W., Zeng F., Wang Y., Liu X., Mebrahtu C., Pei G., Cao J. P., Yao T., Wang Z., Zeng J. (2025). A Scenario for a Carbon-Neutral Ammonia-Fueled
Engine Mediated by Catalytic NH_3_ Cracking and CO_2_ Hydrogenation. Angew. Chem. Int. Ed.

[59] Hanna T. E., Lobkovsky E., Chirik P. J. (2004). Dinitrogen Activation by Titanium
Sandwich Complexes. J. Am. Chem. Soc.

[60] Alkan B., Duarte-Correa L., Girgsdies F., Koch G., Kröhnert J., Ertegi M., Jiang S., Lunkenbein T., Trunschke A. (2025). From “Single Sites” to Stable Nanoparticles
Derived from Spray-Flame Synthesized Solid Solutions of Cobalt in
MgO for Ammonia Decomposition. ACS Catal.

