# Supplementary material for: The Role of BaCO3 on Ni-BaZrO3 Catalysts for Efficient NH3 Decomposition
Source: ACS Omega. 2026 Oct 13;11(42):65397–410. doi: 10.1021/acsomega.6c08031 (PMC13718222; doi:10.1021/acsomega.6c08031)
Supplement: Supplementary file 1 [file ao6c08031_si_001.pdf]

Supplementary Information

The role of $BaCO_3$ on Ni-$BaZrO_3$ catalysts for efficient NH_3 Decomposition

*Andrea Felli[a], Maila Danielis[a], Christine Artner-Wallner[b], Giovanni Capurso[a], Alessandro Trovarelli[a], and Sara Colussi[a]**

[a]Polytechnic Department, University of Udine, Via del Cotonificio 108, 33100, Udine, Italy.

[b]Treibacher Industrie AG, Auer-von-Welsbach-Straße 1, 9330, Althofen, Austria.

*sara.colussi@uniud.it

CHARACTERIZATION OF Ni-$BaZrO_3$ CATALYSTS

Elemental composition analysis through XRF was performed on bare supports ($BaZrO_3$). 10 g of sample were analyzed with a Malvern Panalytical Zetium XRF spectrometer. The resulting data were acquired and analyzed with the Panalytical SuperQ software with an internal databank.

Surface area and pore size analyses were carried out by evaluating N_2 adsorption/desorption isotherms at -196 °C in a Micromeritics Tri-Star 3000 apparatus. 250 mg of sample were firstly degassed below 100 mTorr at 150 °C for 1.5 h to remove adsorbed contaminants and then cooled to -196 °C under vacuum before N_2 introduction. Surface area and pore size distributions were obtained from adsorption and desorption data following the Brunauer-Emmett-Teller theory (BET) and Barrett-Joyner-Halenda method (BJH), respectively.

X-ray diffraction (XRD) characterization was carried out with a Philips PW3040/60 X'Pert PRO diffraction system (with Bragg-Brentano geometry) using Cu Kα radiation (40 kV, 40 mA, λ=0.154 nm). Data were acquired at 0.02° at a counting time of 40 s step^{-1} in the 20-80° 2θ range. Online databases were used for the identification of the crystalline phases. The average crystalline size was calculated by the Scherrer equation.[1] A stainless-steel chamber with Be windows was assembled on a spectrometer to study the phase evolution in a reducing atmosphere at high temperatures, and to study CO_2 exposure; the spectra were acquired at 0.01° steps at a counting time of 80 s step^{-1} in the 2θ range from 20 to 40°.

Thermogravimetric analysis (TGA) was performed using a TGA 5500 (Waters TA Instruments). 20 mg of sample were weighted in a platinum pan and then exposed to air (25 mL min^{-1}); the weight change was recorded while increasing the temperature of the furnace (10 °C min^{-1}) up to 550 °C.

IR spectroscopy by ATR was carried out on a Fourier transform-infrared (FT-IR) spectrometer (Thermo Scientific Nicolet iS-50 ATR) equipped with a deuterated triglycine sulfate (DTGS) detector with KBr windows and a diamond ATR crystal. After the background acquisition, samples were compressed on the crystal and analyzed by acquiring 32 scans between 400-4000 cm^{-1} with a resolution of 4 cm^{-1}.

An Xplora Plus Micro-Raman (Horiba) system was used for Raman spectra collection. Samples were excited with the 532 nm radiation through a 50x LWD objective and a grating of 400 nm, and several filters were also used to optimize the signal. The spectra were acquired with a resolution of 1 cm^{-1} and the number of accumulations, and the scan time were changed considering the type of material analyzed. *In situ* characterization was done using the same conditions, using a stainless steel chamber with a quartz window, flowing 50 mL min^{-1} of a 5%H_2/N_2 mixture, and acquiring spectra every 100 °C.

A field-emission-gun scanning electron microscope (FEG-SEM, Jeol JSM-7600F) equipped with energy dispersive X-ray spectroscopy (EDS, Oxford X-Max) was used for the acquisition of micrographies, using secondary electrons, and elemental maps to further analyze and characterize the samples. Images were collected at an acceleration voltage ranging between 15 kV and magnifications at 80’000. The samples were sputter-coated with a very thin (2–5 nm) layer of Au to enhance the electrical conductivity.

H_2-TPR experiments were carried out in a Micromeritics AutoChem II 2920 analyzer equipped with a TCD detector. The sample was loaded in a U-shaped quartz microreactor on a quartz wool bed. 50 mg of sample were pretreated in air (35 mL min^{-1}) at 350 °C (10 °C min^{-1}) for 1 h and then cooled to 50 °C in pure N_2. The gas feed was then switched to the reducing mixture (5% H_2 in N_2, 35 mL min^{-1}), and the temperature was ramped up to 900 °C (10 °C min^{-1}). H_2 concentration was

measured with a TCD detector. Additional H_2-TPR tests were carried out in a tubular quartz reactor, loading 100 mg of sample under 50 mL min^{-1} of a 5%H_2/N_2 mixture. H_2, CH_4, CO_2 and CO concentrations were monitored every 10s using an ABB Optima AO2020 Gas analyzer equipped with a Caldos 27 and a Uras 26 module, respectively.

CO_2-TPD experiments were carried out by pretreating the samples in 5% of H_2 in He (35 mL min^{-1}) at 550 °C (10 °C min^{-1}) for 1 h and then cooled to 60 °C in pure He. The sample was then kept at 60 °C while feeding 20% of CO_2 in He (35 mL min^{-1}) for 1 h. The gas feed was then switched to 35 mL min^{-1} of He and the temperature was increased up to 900 °C at a heating rate of 10 °C min^{-1}. During temperature ramp, CO_2 desorption was followed by a GSD 350 Omnistar mass spectrometer (Pfeiffer vacuum).

In situ diffuse reflectance infrared Fourier transform spectroscopy (DRIFTS) experiments mimicking both CO_2 TPD and ammonia decomposition were carried out in a Thermo Scientific Nicolet iS-50 FTIR equipped with a deuterated triglycine sulfate (DTGS) detector and a stainless-steel hot chamber (Pike DiffuseIR). For CO_2-TPD analysis, *ex situ* reduced samples (at 550 °C for 1h in H_2) were pretreated again at 400 °C to ensure surface cleaning and then exposed to 20% of CO_2 in He (100 mL min^{-1}) for 1 h. The samples were then switched in N_2 and heated at 450 °C. Spectra were acquired every 10 °C. NH_3 decomposition was carried out in the stainless-steel hot chamber (Pike DiffuseIR) flowing NH_3 (2.5% in N_2, 80 mL min^{-1}) on the sample previously pretreated at 550 °C in 5% of H_2 in N_2 (40 mL min^{-1}) for 1 h. Spectra were acquired at different temperatures up to 480 °C reproducing the same procedure of NH_3 decomposition tests. For both type of experiments, the background was collected at the corresponding temperatures in pure N_2.

Temperature-programmed surface reaction (TPSR) experiments were carried out in the same setup of NH_3 decomposition tests. 100 mg of sample were pretreated in 5% of H_2 in He (40 mL

min^{-1}) from room temperature up to 550 °C (10 °C min^{-1}) for 1 h and then cooled down in pure He at 480 °C. The sample was then exposed to 5.0% of NH_3 in He (30 mL min^{-1}, GHSV ≈ 15’000 h^{-1}) for 1h and cooled down in pure He at 250 °C. The catalytic bed was then heated until 900 °C. NH_3, N_2, and H_2 were monitored by a GSD 350 Omnistar mass spectrometer (Pfeiffer vacuum).

MORPHOLOGICAL PROPERTIES OF SYNTHESIZED $BaZrO_3$ AND Ni-$BaZrO_3$

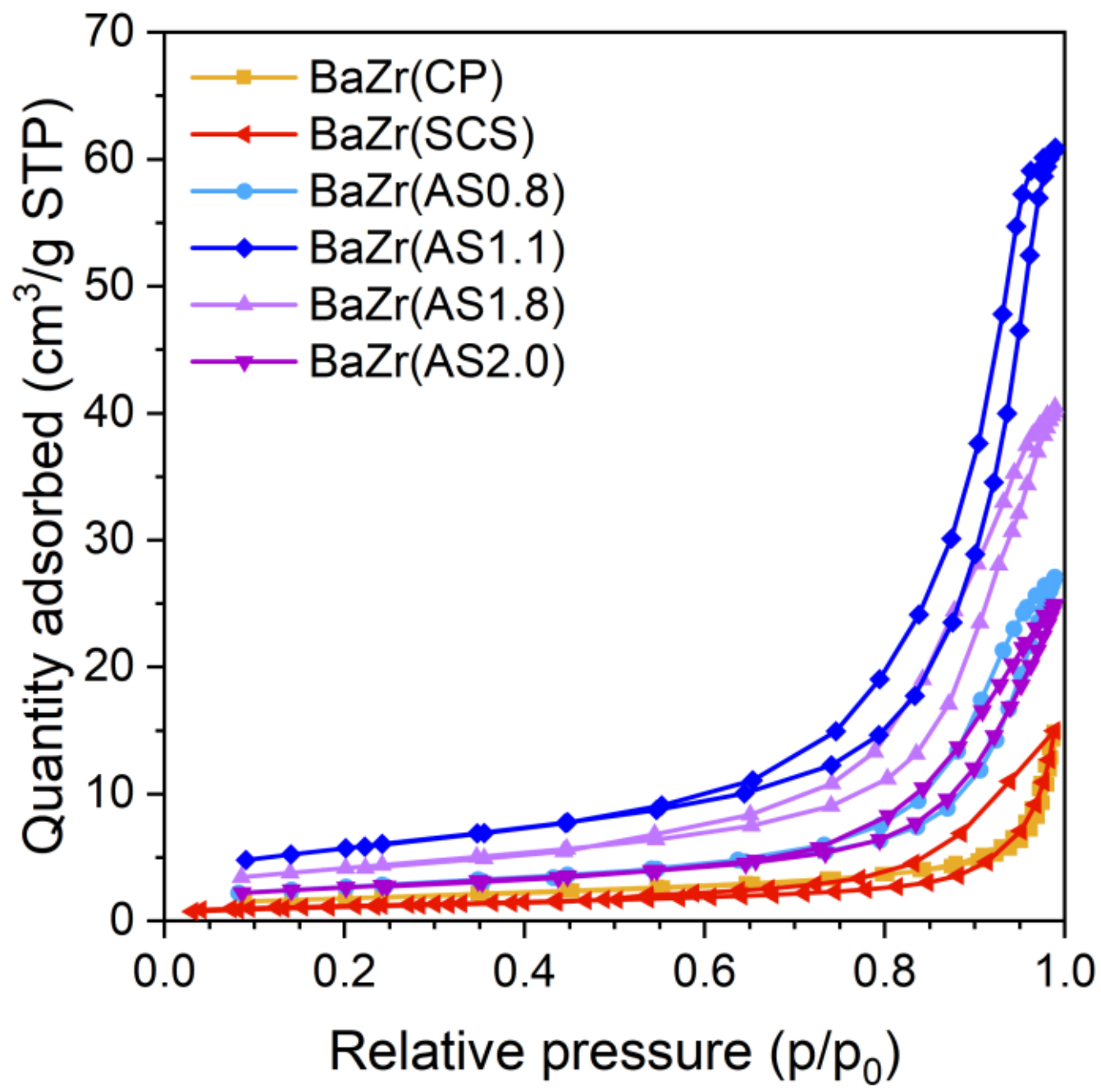

Figure S1. N_2 isotherms of the most representative $BaZrO_3$ samples.

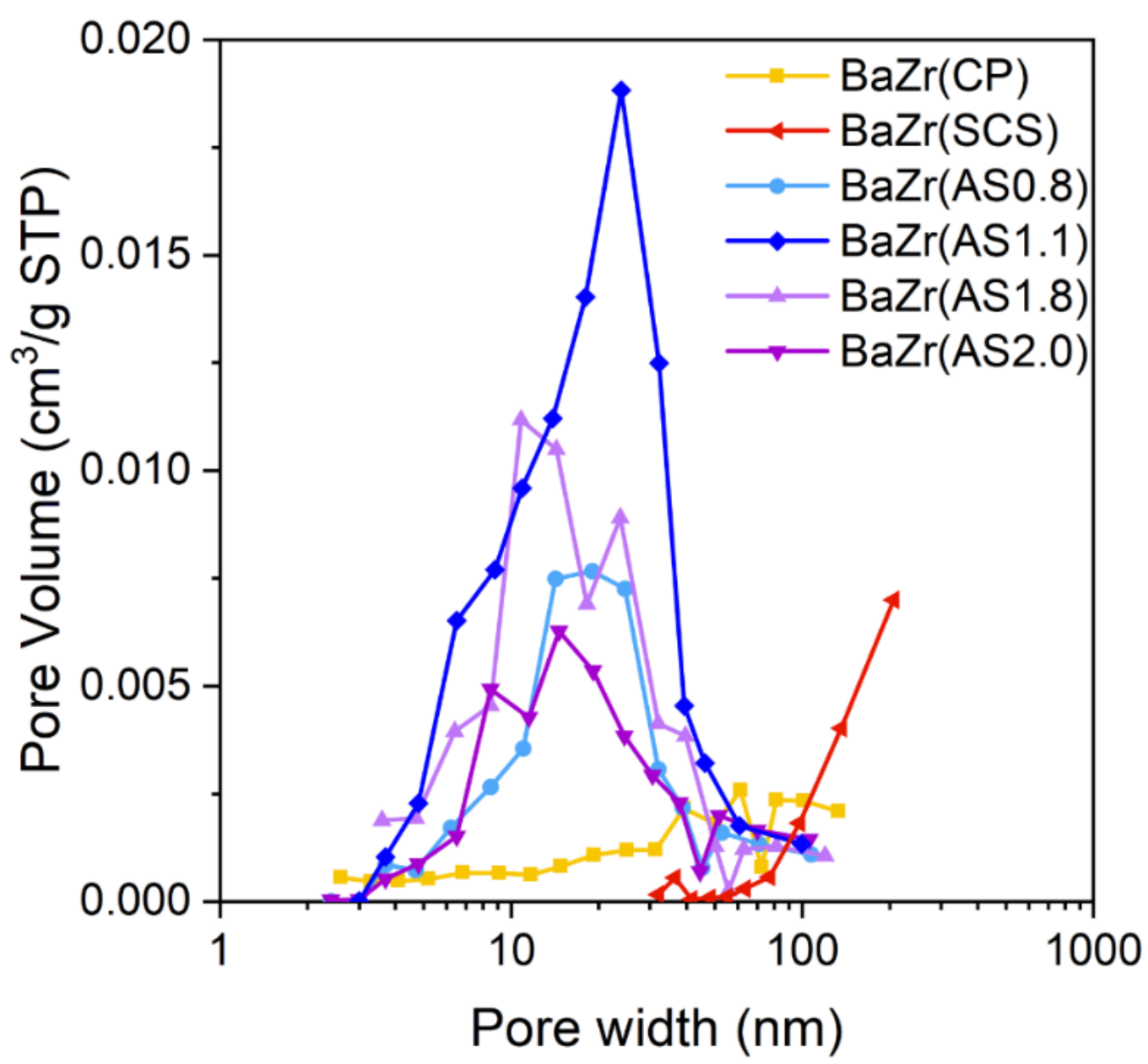

Figure S2. Pore size distribution of the most representative $BaZrO_3$ samples.

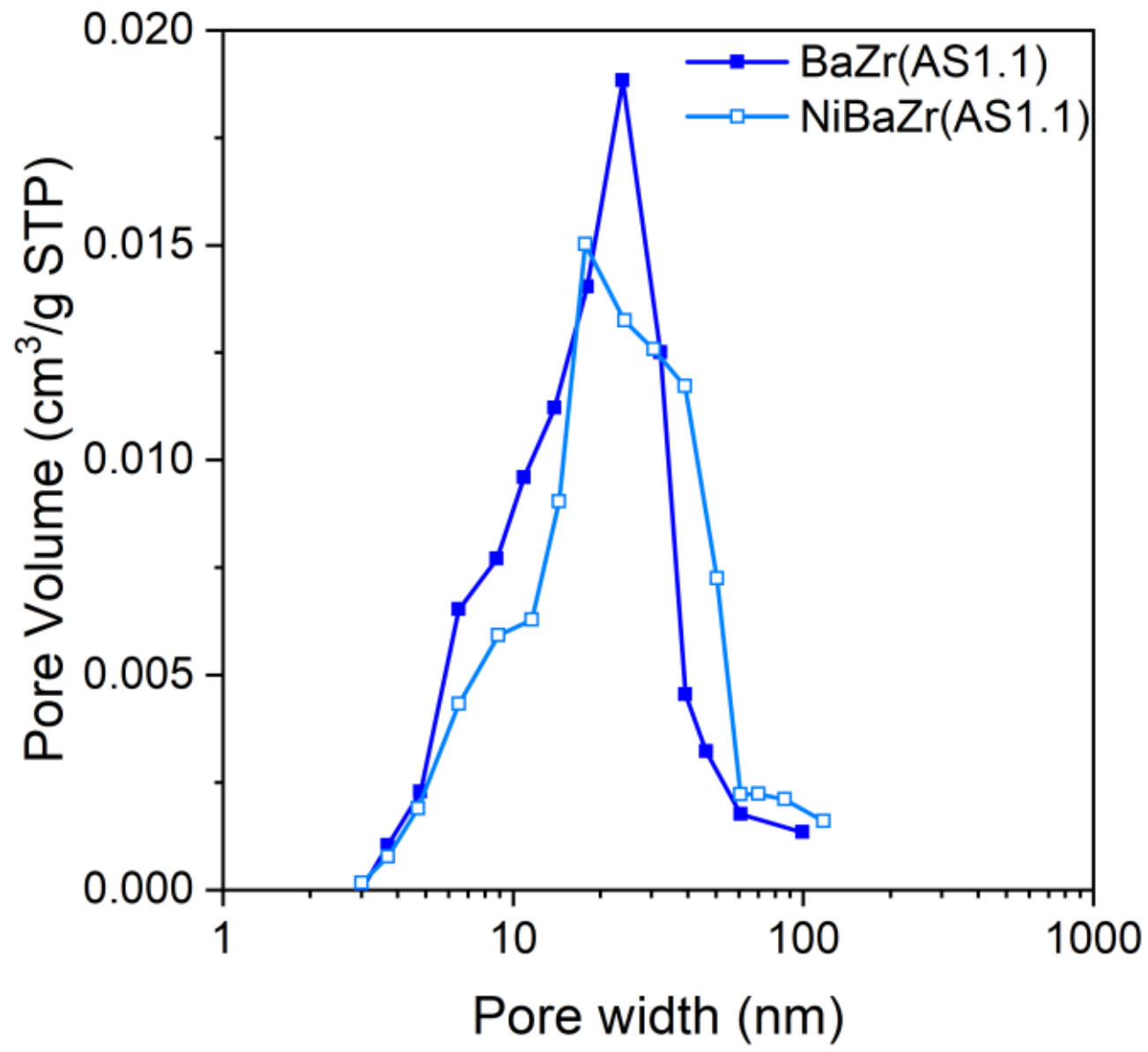

Figure S3. Pore size distribution of BaZr(AS1.1) and NiBaZr(AS1.1).

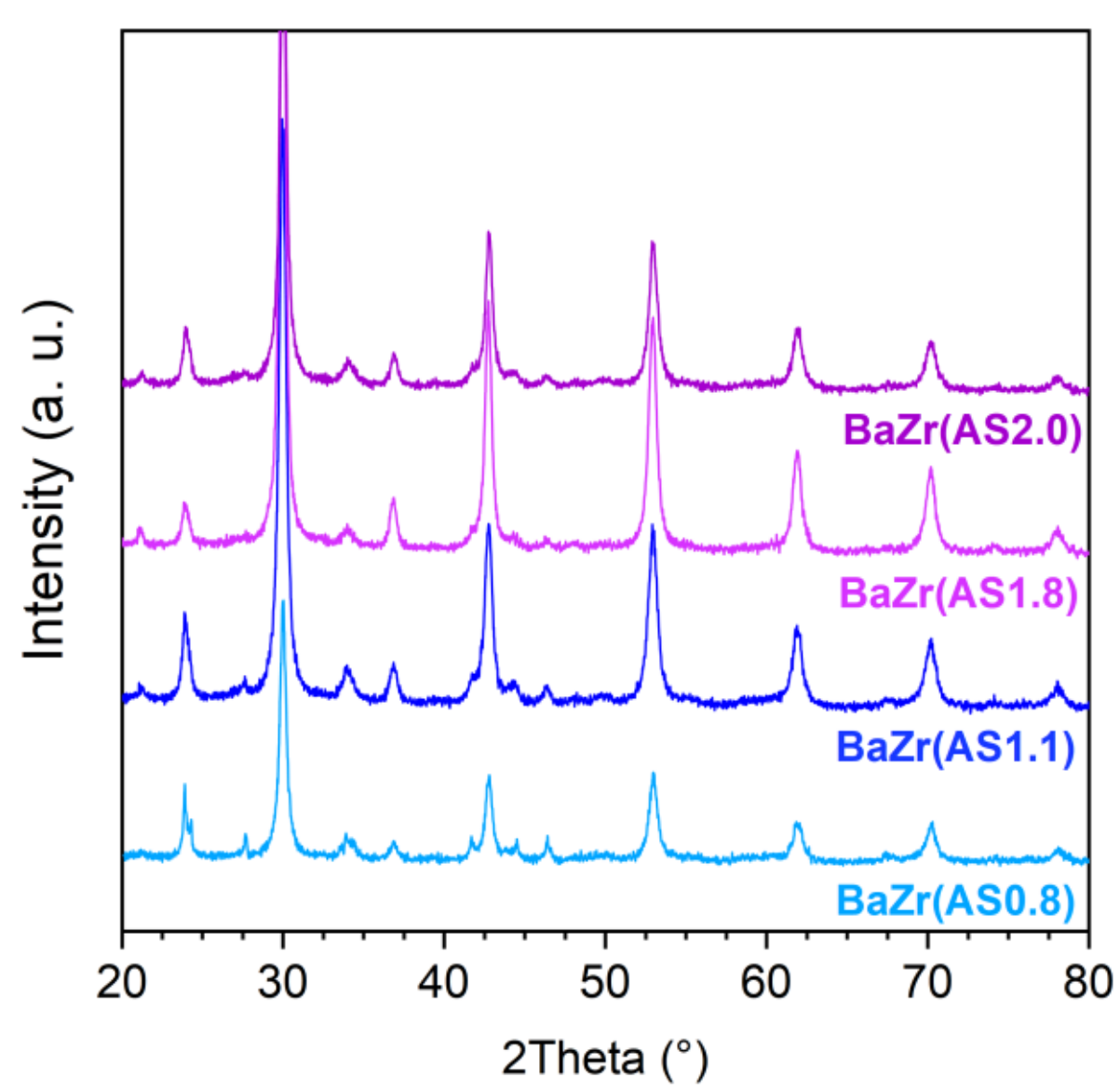

Figure S4. X-ray diffractograms of BaZr(AS0.8), (AS1.1), (AS1.8), and (AS2.0).

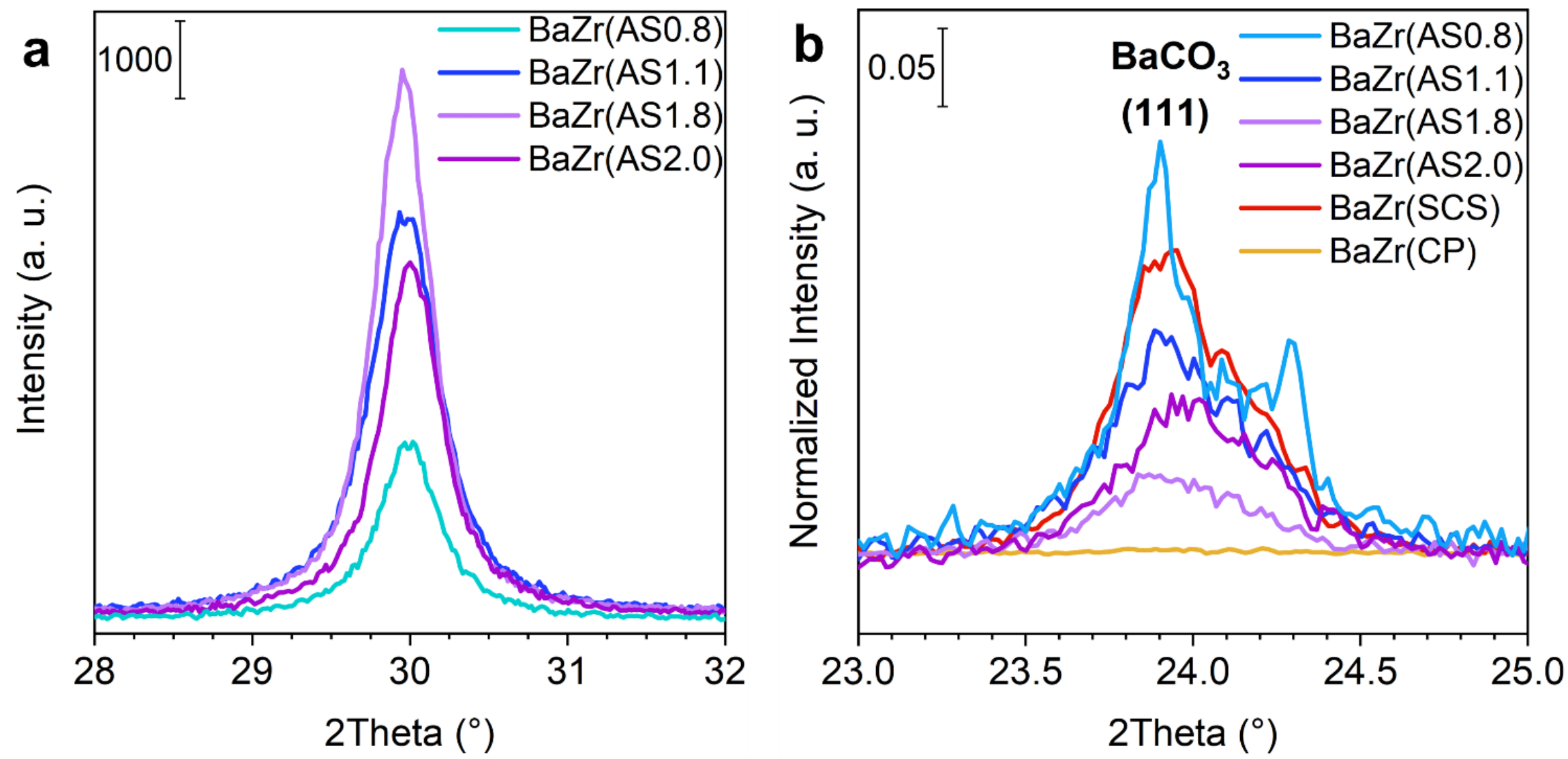

Figure S5a. Magnification between 28-32° 2θ on (110) signal of BaZr(AS0.8), (AS1.1), (AS1.8), and (AS2.0); **b.** Magnification between 23-35° 2θ on (111) signal of $BaCO_3$ for BaZr(CP), BaZr(SCS), and BaZr(AS0.8), (AS1.1), (AS1.8), and (AS2.0); signal normalized on the main $BaZrO_3$ peak at 30° 2θ.

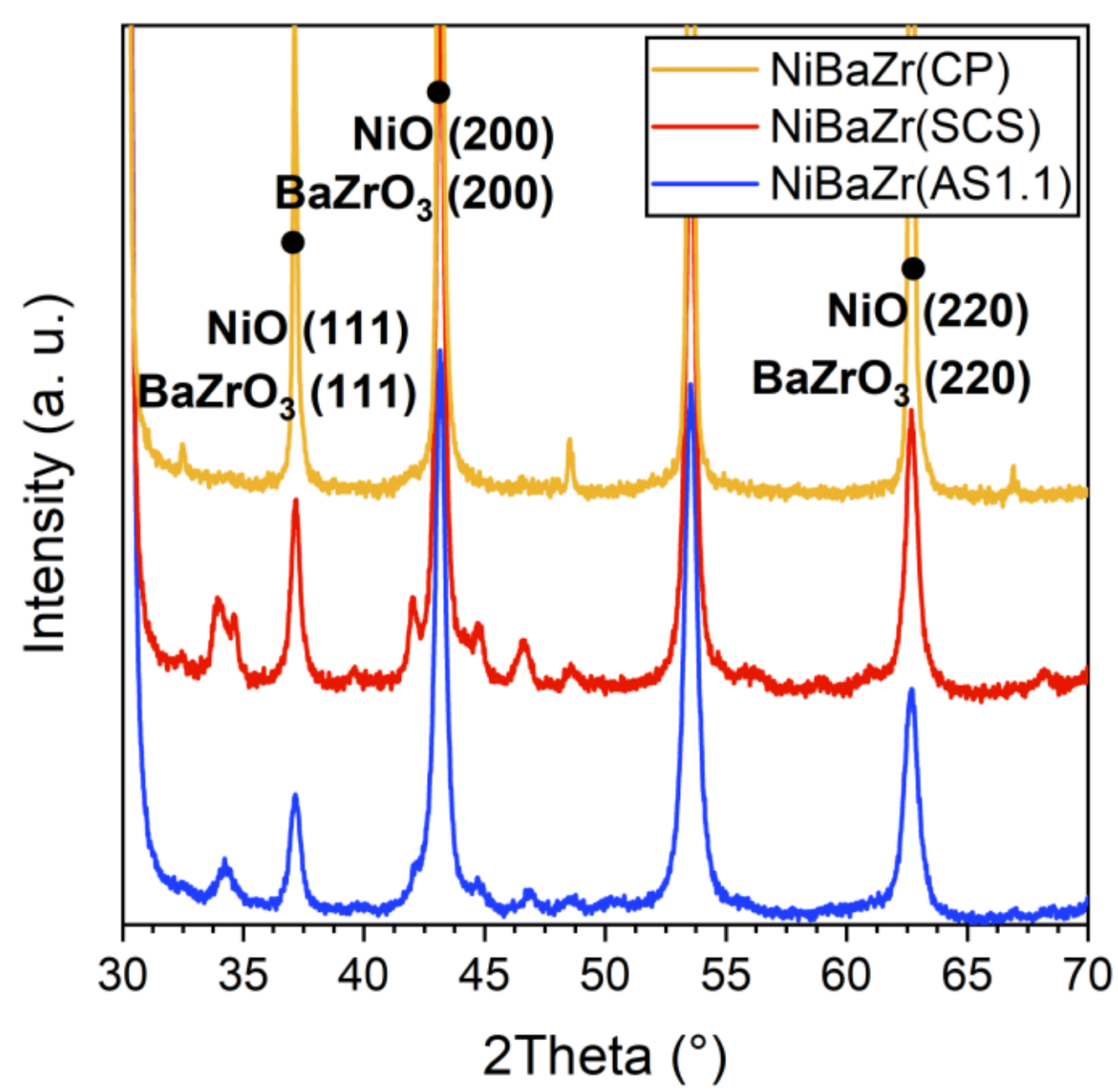

Figure S6. X-ray diffractograms of NiBaZr(CP), NiBaZr(SCS), and NiBaZr(AS1.1).

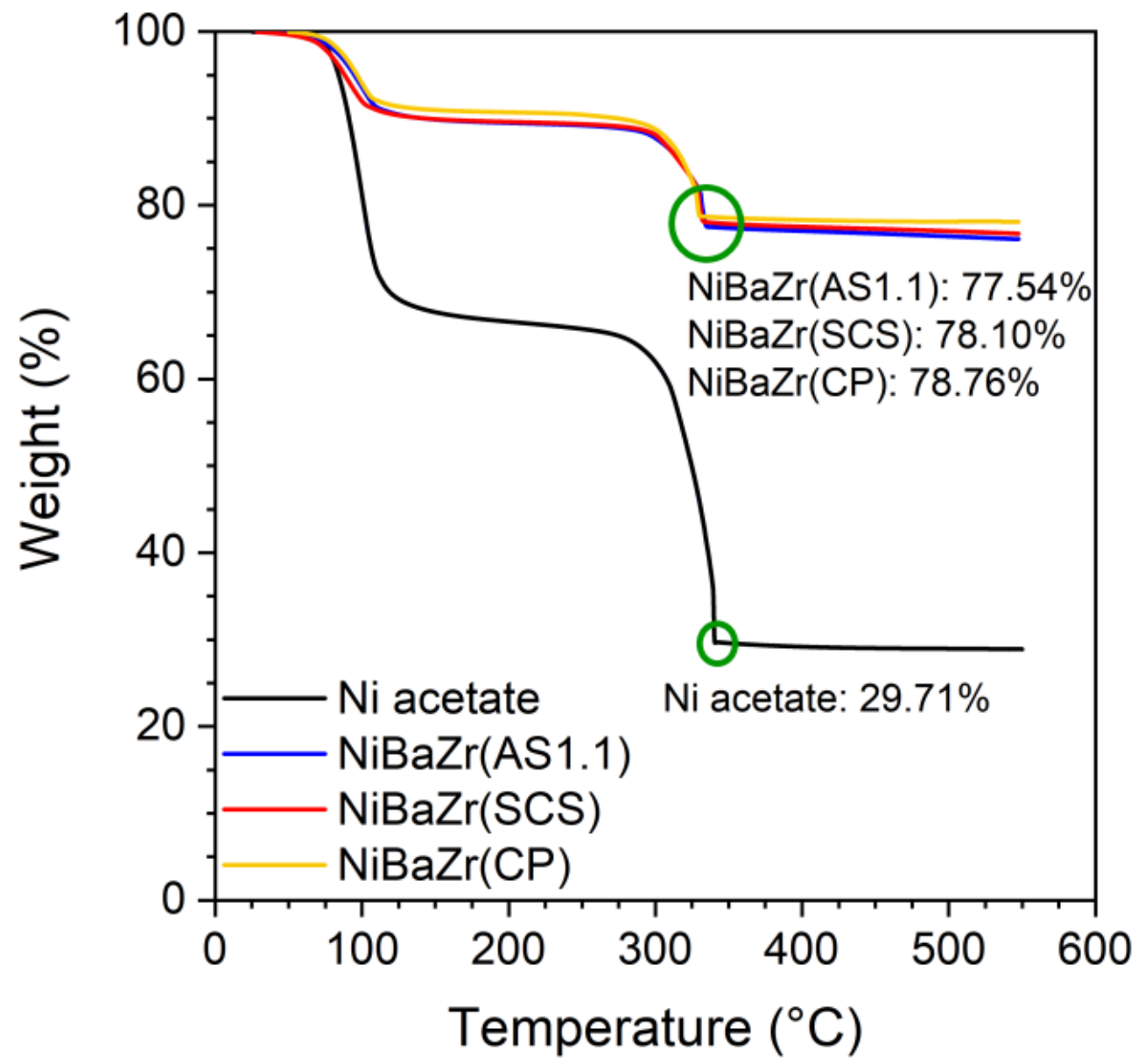

Figure S7. TG analysis of Ni acetate and Ni acetate deposited on BaZr(AS1.1), BaZr(SCS), and BaZr(CP).

The formation of NiO can be inferred from TGA analysis (**Figure S7**). According to literature,[2] Ni acetate decomposes in air in two steps, with complete decomposition at 343 °C and an observed residual weight of 29.71%, consistent with NiO formation (theoretical: 30.01%). TGA of Ni–$BaZrO_3$ samples shows similar behavior, with residual weights at 343 °C of 77.54% (AS1.1), 78.10% (SCS), and 78.76% (CP), in line with the expected value of 12.73% of NiO (corresponding to 10% Ni) formation on $BaZrO_3$ (80.69%). Minor deviations are attributed to the release of adsorbed species (CO_2, H_2O) from the support at low temperatures.

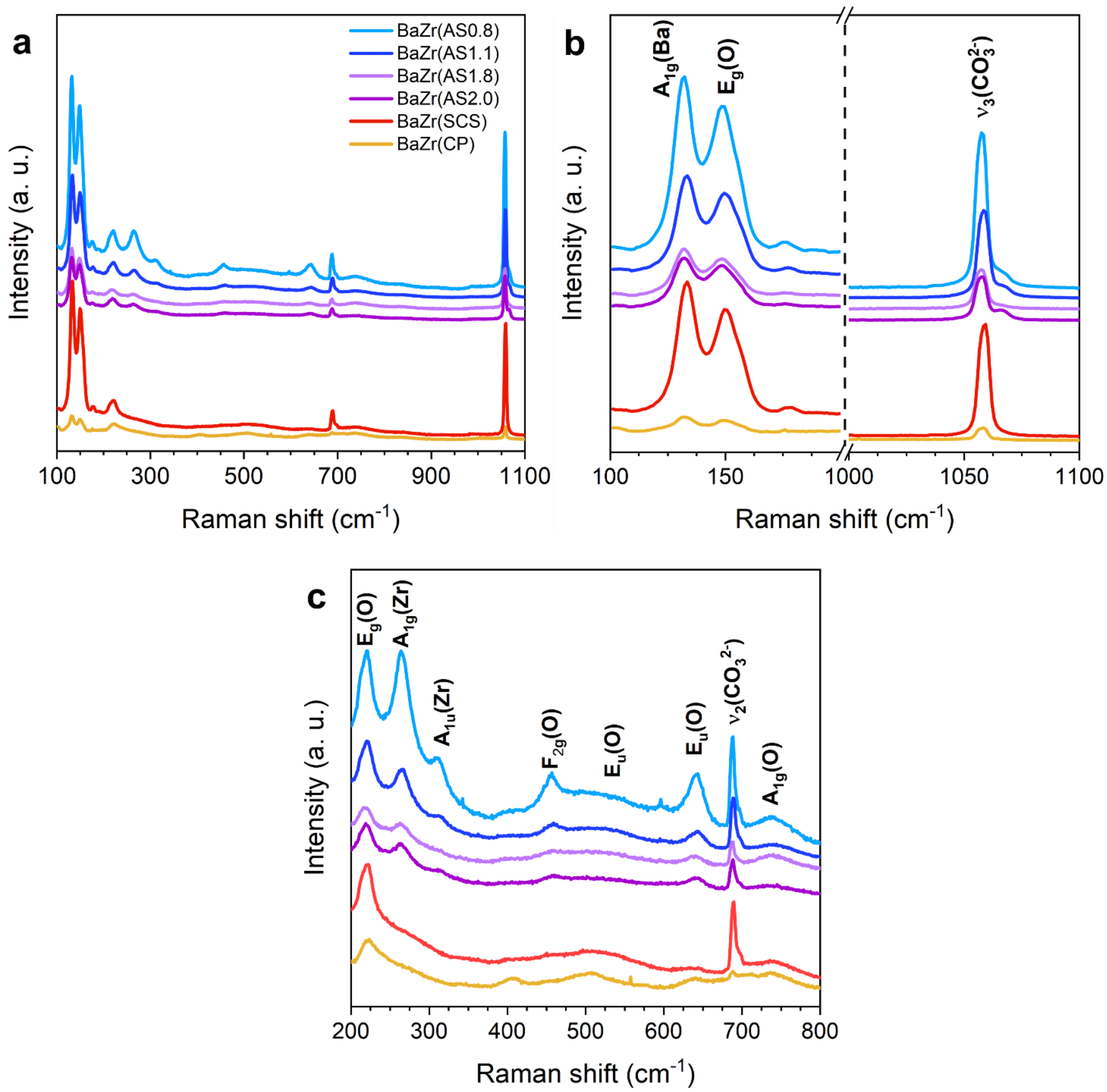

Figure S8a. Raman spectra of $BaZrO_3$ CP, $BaZrO_3$ SCS, and $BaZrO_3$ AS 0.8, 1.1, 1.8, and 2.0; **b.** magnification between 100-200 cm^{-1} on the A_{1g} and the E_g signals and between 1000-1100 cm^{-1} on $\nu_3(CO_3^{2-})$ signal; **c.** magnification on the low to medium frequencies area.

Table S1. Raman peaks attribution and frequencies for BaZr(CP), BaZr(SCS), and BaZr(AS1.1).

Raman peak attribution	Frequency (cm^{-1})			
	BaZr(AS 1.1)	BaZr(CP)	BaZr(SCS)	Ref.
A_{1g}(Ba): lattice vibration of Ba atoms in $BaCO_3$	132	133	133	3
E_g(O): lattice vibration of O atoms in $BaCO_3$	149	150	150	3
E_g(O): symmetric vibrations of Zr-O-Zr into the lattice	221	222	221	3
A_{1g}(Zr): symmetric vibrations of Zr-O-Zr into the lattice	267	(267)	(267)	3
A_{1u}(Zr): Zr antiparallel displacement	311	(311)	(311)	4
F_{2g}(O): O atoms breath vibration in the $[ZrO_6]$ octahedra	457	457	457	3
E_u(O): displacement of apical O atoms against in-plane atoms	506	506	506	4
E_u(O): displacement of apical O atoms against in-plane atoms	643	640	642	3
ν_1 (CO_3^{2-}): bending in-plane vibrations	688	688	688	5
A_{1g}(O): breath vibration of O^{2-} in $[ZrO_6]$	742	741	742	3
A_{1g}(O): breath vibration of O^{2-} in $[ZrO_6]$	845	842	841	3
ν_2 (CO_3^{2-}): asymmetric stretching vibrations	1059	1058	1059	5
ν_3 (CO_3^{2-}): symmetric stretching vibrations	1420	-	1420	5

The full Raman spectra (**Figure S8a**) display a more complex pattern than commonly observed for perovskite oxides. Among the AS samples spectral intensity decreases from BaZr(AS0.8) to BaZr(AS2.0). The intensity of bands on SCS sample is comparable to that of BaZr(AS1.1) but some features are lacking, while BaZr(CP) exhibits a much weaker signal. The variations in intensity correlate with $BaCO_3$ content, evident in the lattice translation modes of Ba (133 cm^{-1}) and O (150 cm^{-1}) in carbonate structures (**Figure S8b**),[3,6] mirroring ATR trends. Perovskite-related modes mostly appear between 200 and 800 cm^{-1} (**Figure S8c**). Bands at around 220 cm^{-1} and 270 cm^{-1} correspond to F_{2g}(Zr) split into E_g(Zr) and A_{1g}(Zr) modes, associated with symmetric Zr–O–Zr vibrations.[3] AS samples show a more intense A_{1g}(Zr) peak than the reference samples,

indicating greater distortion of $[ZrO_6]$ octahedra and higher defect concentration, especially at lower CA/C^+ ratios. This is supported by the presence of a band at 311 cm^{-1} (Zr antiparallel displacement),[4] F_{2g}(O) vibration at 457 cm^{-1}, and E_u(O) modes at 500 and 640 cm^{-1}, linked to bulk and surface displaced oxygen atoms.[3,4] Additional A_{1g}(O) vibrations appear at 745 and 842 cm^{-1}, representing oxygen breathing modes in $[ZrO_6]$.

CATALYTIC AMMONIA DECOMPOSITION ACTIVITY

Table S2. Comparison between NiBaZr(AS 1.1) performance for ammonia decomposition with benchmark materials from literature.

Sample	WHSV (mL h^{-1} g_{cat}^{-1})	NH_3 (vol%)	Reaction T (°C)	NH_3 conversion (%)	H_2 production ($mmol_{H2}$ g_{cat}^{-1} min^{-1})	Ref.
NiBaZr(AS1.1)	36,000	5	480	67	1.2	This work
Ni/$BaZrO_3$	6000	100	550	95	/	7
NiCo/$BaZrO_3$	9000	100	550	20	/	8
Ni/BZY	6000	100	550	98	/	9
K-Ni/$BaZrO_3$	30,000	6	450	56	1.0	10
Ni/$LaMnO_3$	20,400	10	505	25	0.5	11
50%Ni/$LaMnO_3$	20,400	10	490	50	1.0	12
0.5%Ru-50%Ni/$LaMnO_3$	20,400	10	485	50	1.0	12
Ni/GNP	15,000	10	475	68.0	1.0	13
Ni-$NaNH_2$/GNP	15,000	10	475	70.0	1.1	13
Ni-$NaNH_2$/GNP	15,000	10	475	80.0	1.2	13
Ni/$Al_{0.1}Ce_{0.9}O_x$	30,000	15	500	45.0	2.1	14
Ni/$Al_{0.1}Ce_{0.9}O_x$-MM	30,000	15	500	27.0	1.2	14
Ni/Al_2O_3	30,000	15	500	12.0	0.5	14
Ni/CeO_2	30,000	15	500	17.0	0.8	14
Ni/Al_2O_3	36’000	33	500	~28	/	15

Table S3. Samples name, specific surface area, and reaction rate of studied Ni-based catalysts.

Sample Name	Supporting oxide	BET SSA (m^2 g^{-1})	Reaction rate at 380°C (μmol g^{-1} s^{-1})
NiBaZr(AS1.1)	$BaZrO_3$	20	1.17
NiBaZr(SCS)	$BaZrO_3$	4	0.52
NiBaZr(CP)	$BaZrO_3$	4	0.35
NiAl	Al_2O_3	106	0.43
NiZr	ZrO_2	77	0.45
NiCe	CeO_2	62	0.30

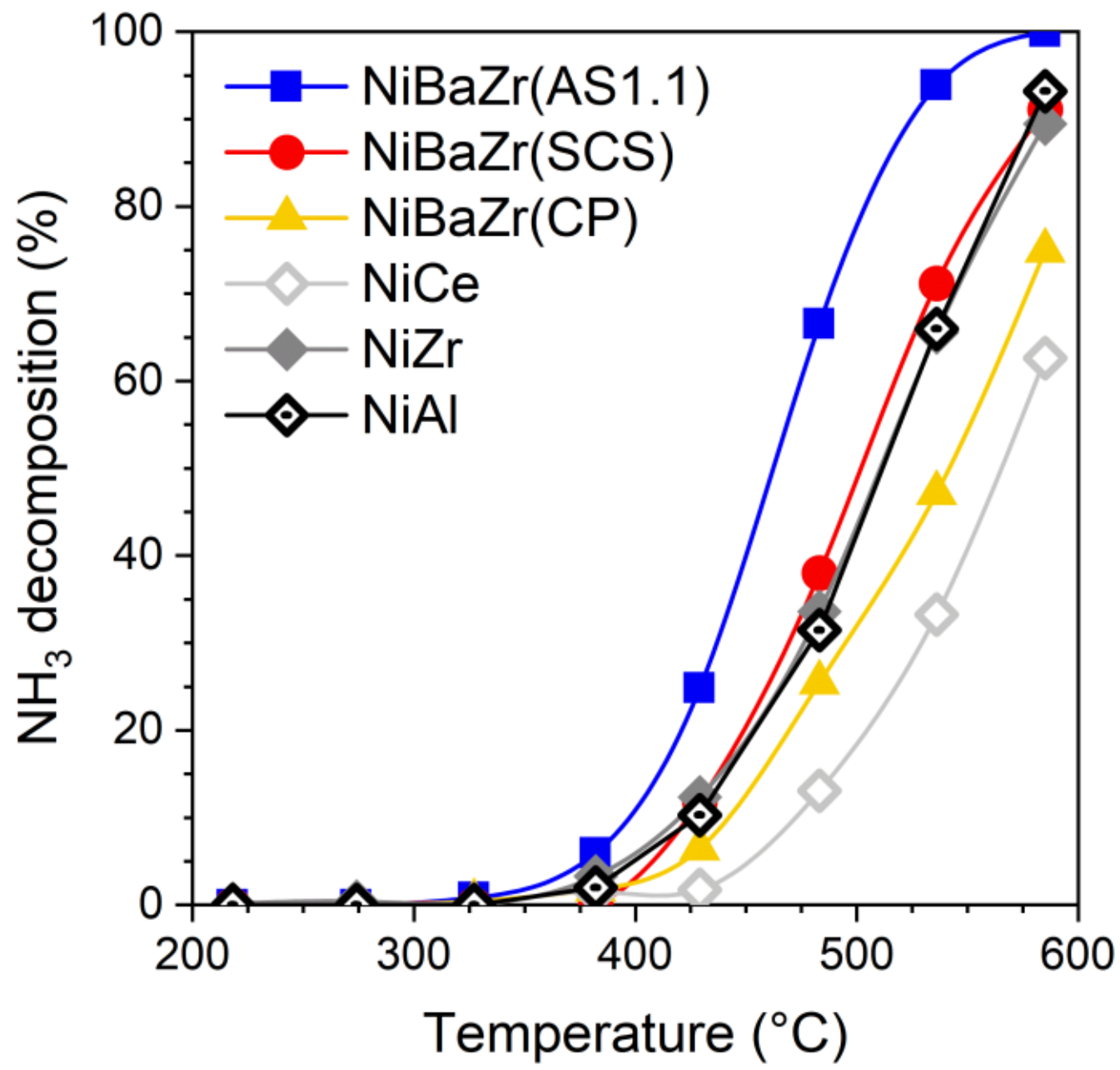

Figure S9. Light-off curves of NiBaZr(CP), NiBaZr(SCS), NiBaZr(AS1.1), NiCe, NiZr, and NiAl catalysts for ammonia decomposition reaction (GHSV~30'000 h^{-1}).

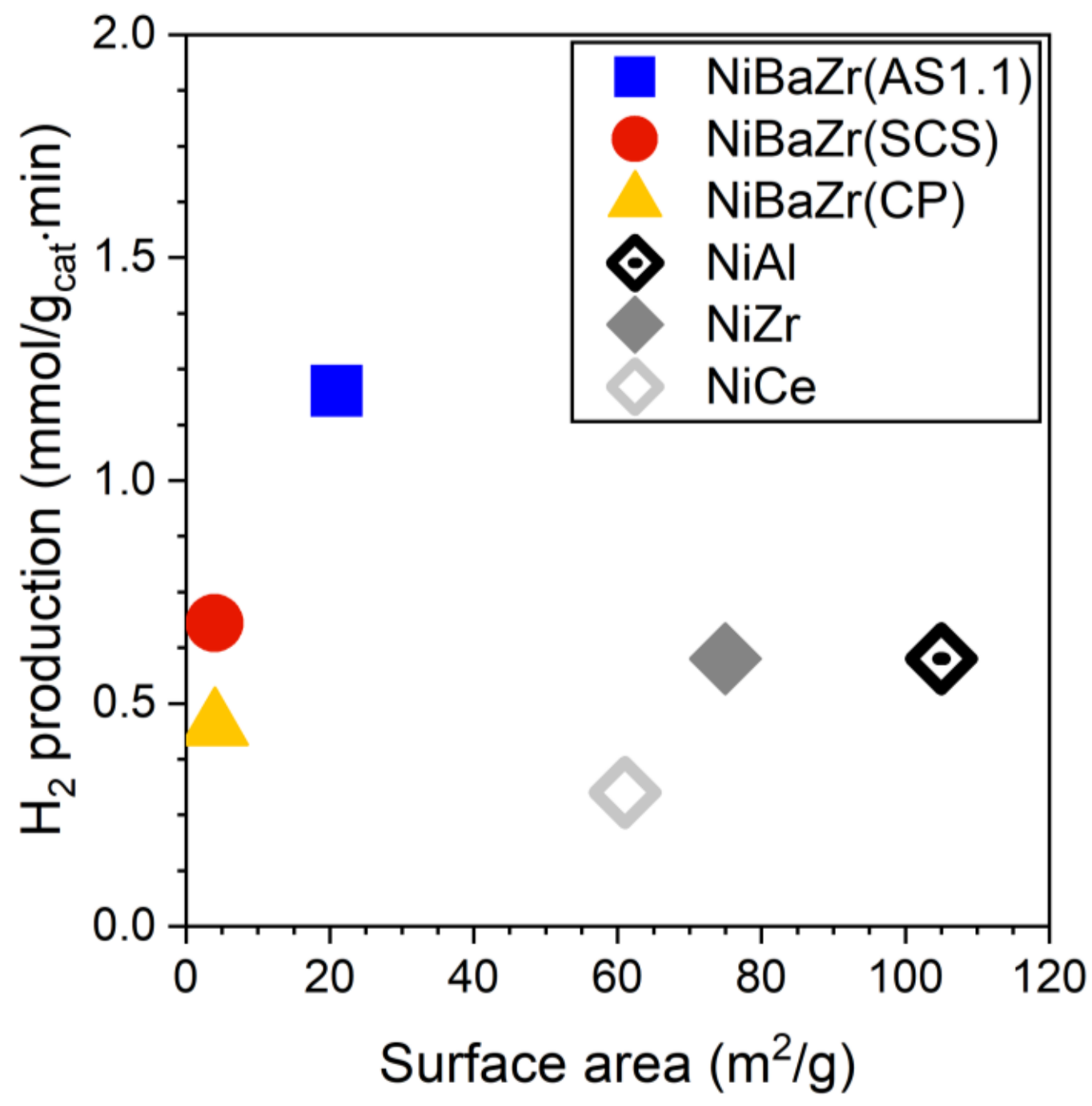

Figure S10. H_2 production at 480 °C (GHSV~30'000 h^{-1}) as a function of the specific surface area for the selected Ni-based catalysts.

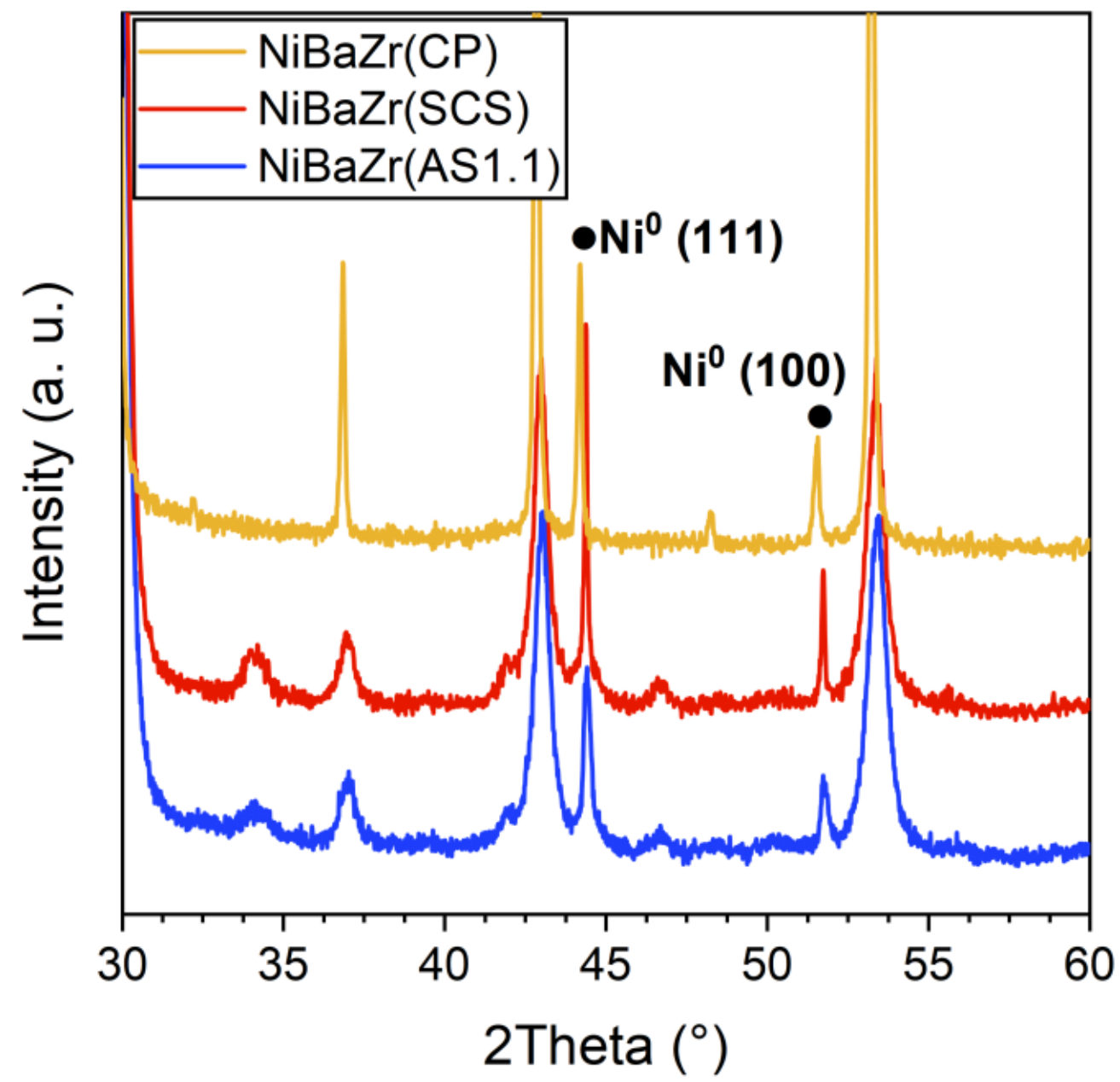

Figure S11. X-ray diffractograms of NiBaZr(CP), NiBaZr(SCS), and NiBaZr(AS1.1) after NH_3 decomposition test. ●: Ni^0.

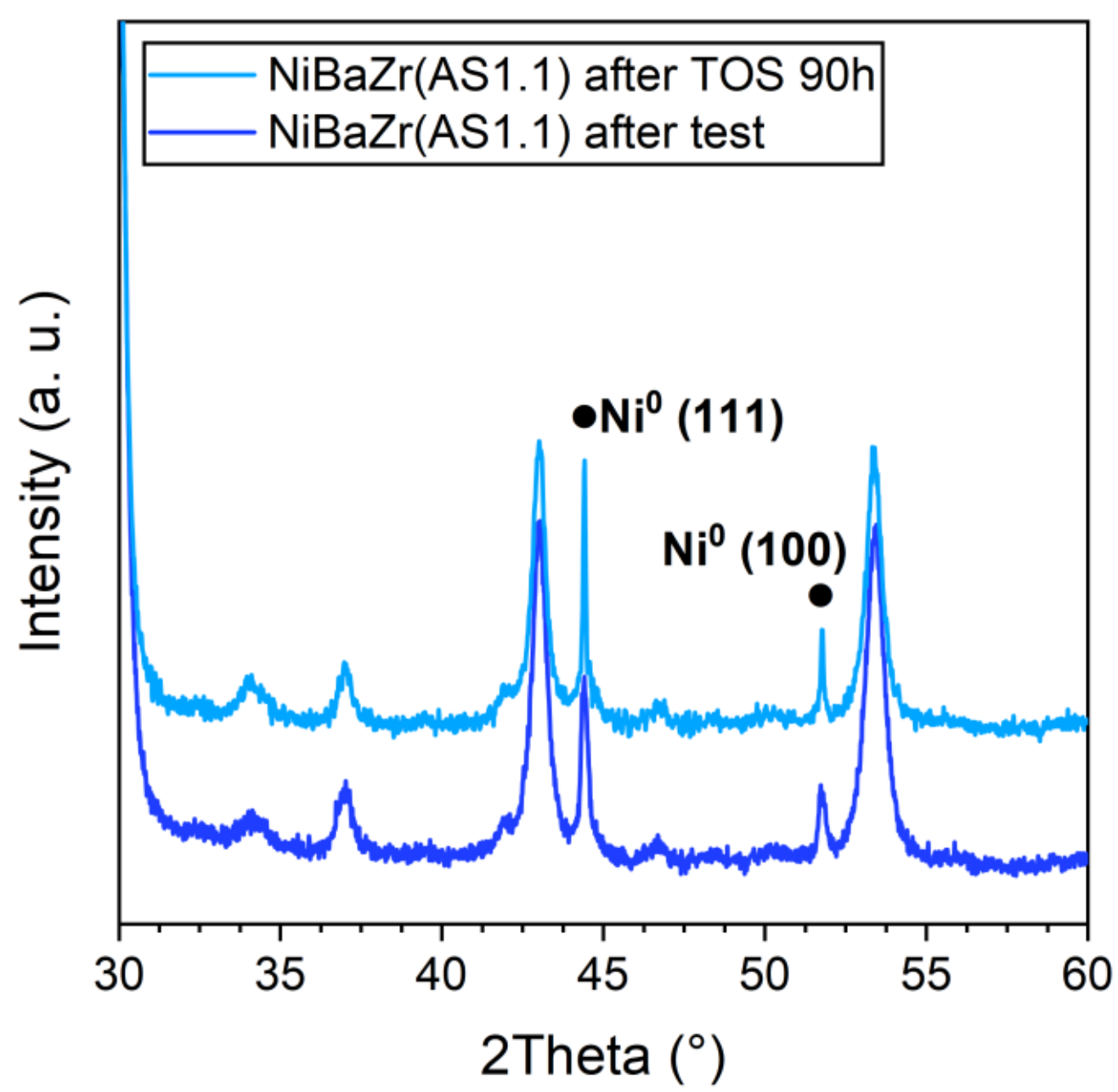

Figure S12. X-ray diffractograms of NiBaZr(AS1.1) after NH_3 decomposition test (shown in **Figure 2a**) and after time-on-stream (TOS) test (reported in **Figure 2c**). ●: Ni^0.

REDOX AND SURFACE PROPERTIES

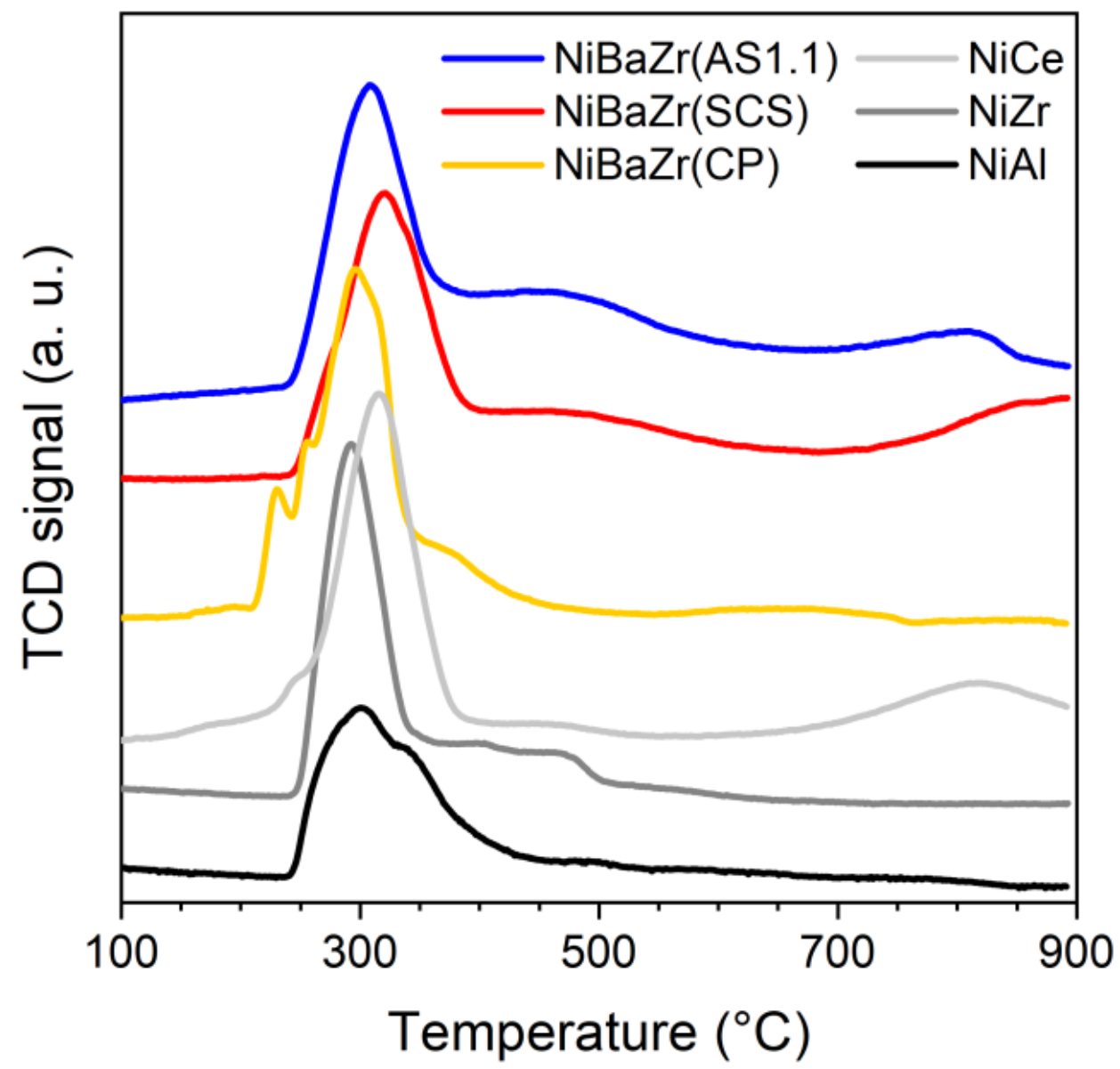

Figure S13. TPR analysis of NiBaZr(CP), NiBaZr(SCS), NiBaZr(AS1.1), NiCe, NiZr, and NiAl catalysts.

Table S4. Quantitative analysis of gaseous species consumed and produced during reduction of BaZr(AS1.1) and NiBaZr(AS1.1).

		Sample					
		BaZr(AS1.1)			NiBaZr(AS1.1)		
**T range (°C)**		**30-550**	**550-800**	**30-800**	**30-550**	**550-800**	**30-800**
**H_2 consumption (µmol g^{-1})**		106	409	515	2857	390	3247
**Gas formation (µmol g^{-1})**	**CO**	76	298	374	26	209	235
	**CH_4**	/	/	/	216	37	253
	**CO_2**	83	9	92	120	18	138
**Excess of H_2 consumption* (µmol g^{-1})**		30	111	141	267	34	301

* Calculated with respect to the theoretical H_2 consumption, estimated with the following reactions (S1-S3) and assuming 100% NiO reduction between 30-550 °C.

S1) $$BaCO_3 + H_2 \rightarrow BaO + CO + H_2O$$

S2) $$BaCO_3 + 4H_2 \rightarrow BaO + CH_4 + 2H_2O$$

S3) $$NiO + H_2 \rightarrow Ni + H_2O$$

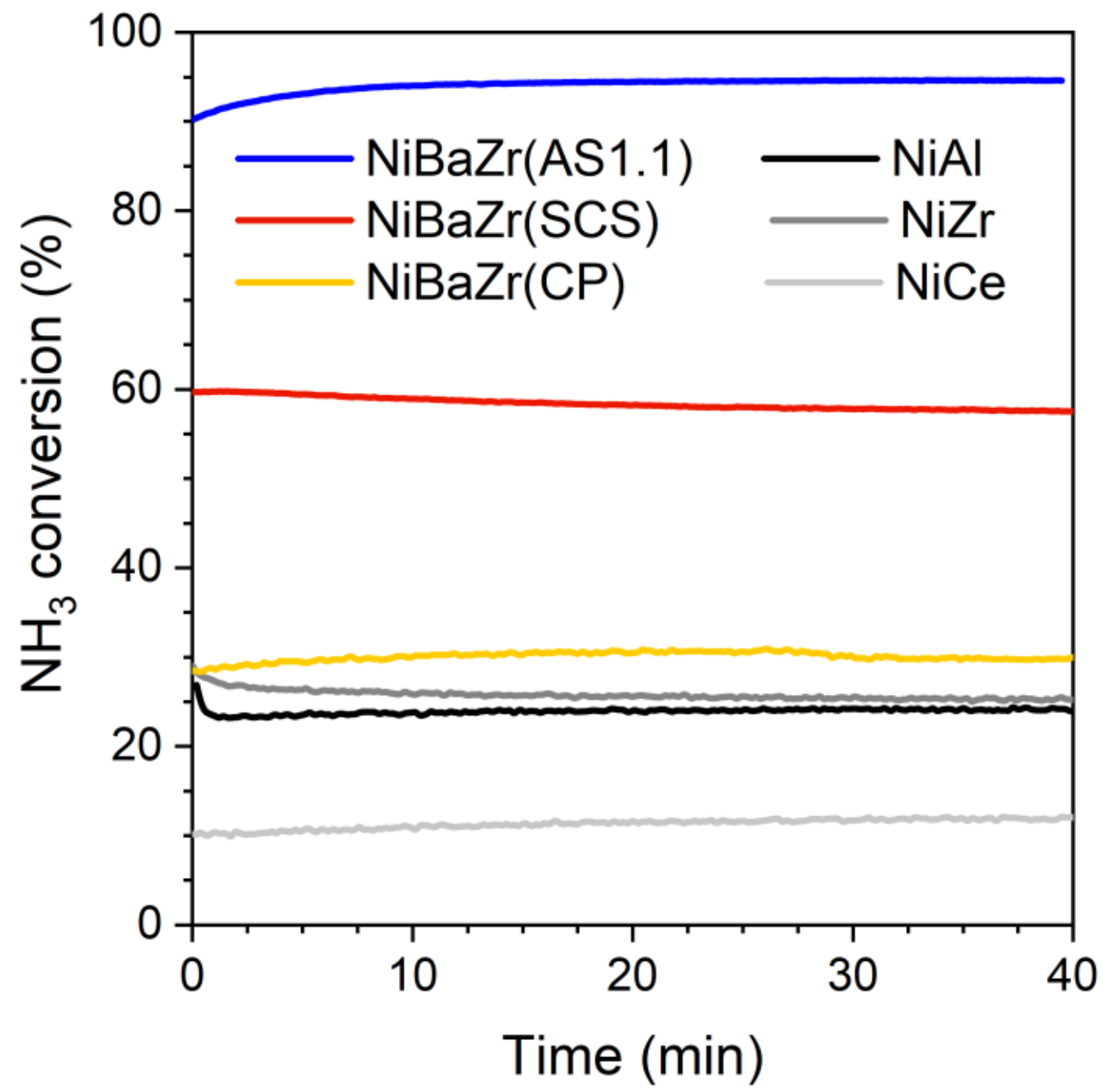

Figure S14. Ammonia decomposition at 480°C for 40 min before the TPSR test (100 mg, 30 mL min^{-1}, GHSV~15’000 h^{-1}).

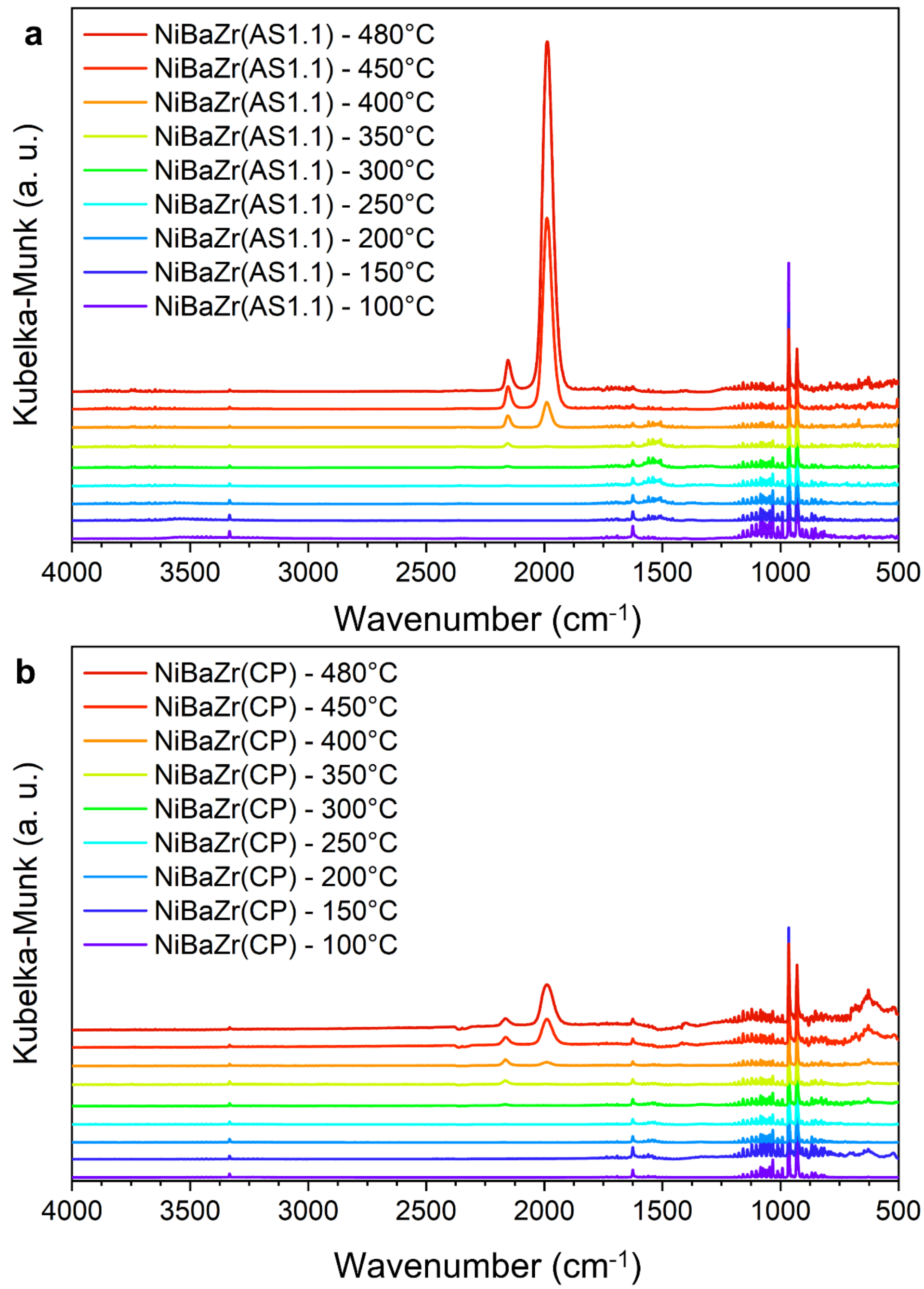

Figure S15. Full spectra collected during DRIFTS analysis of ammonia decomposition reactivity of: **a.** NiBaZr(AS1.1); **b.** NiBaZr(CP).

CONSIDERATIONS ON ACETATE SYNTHESIS AND ITS ENVIRONMENTAL IMPACT

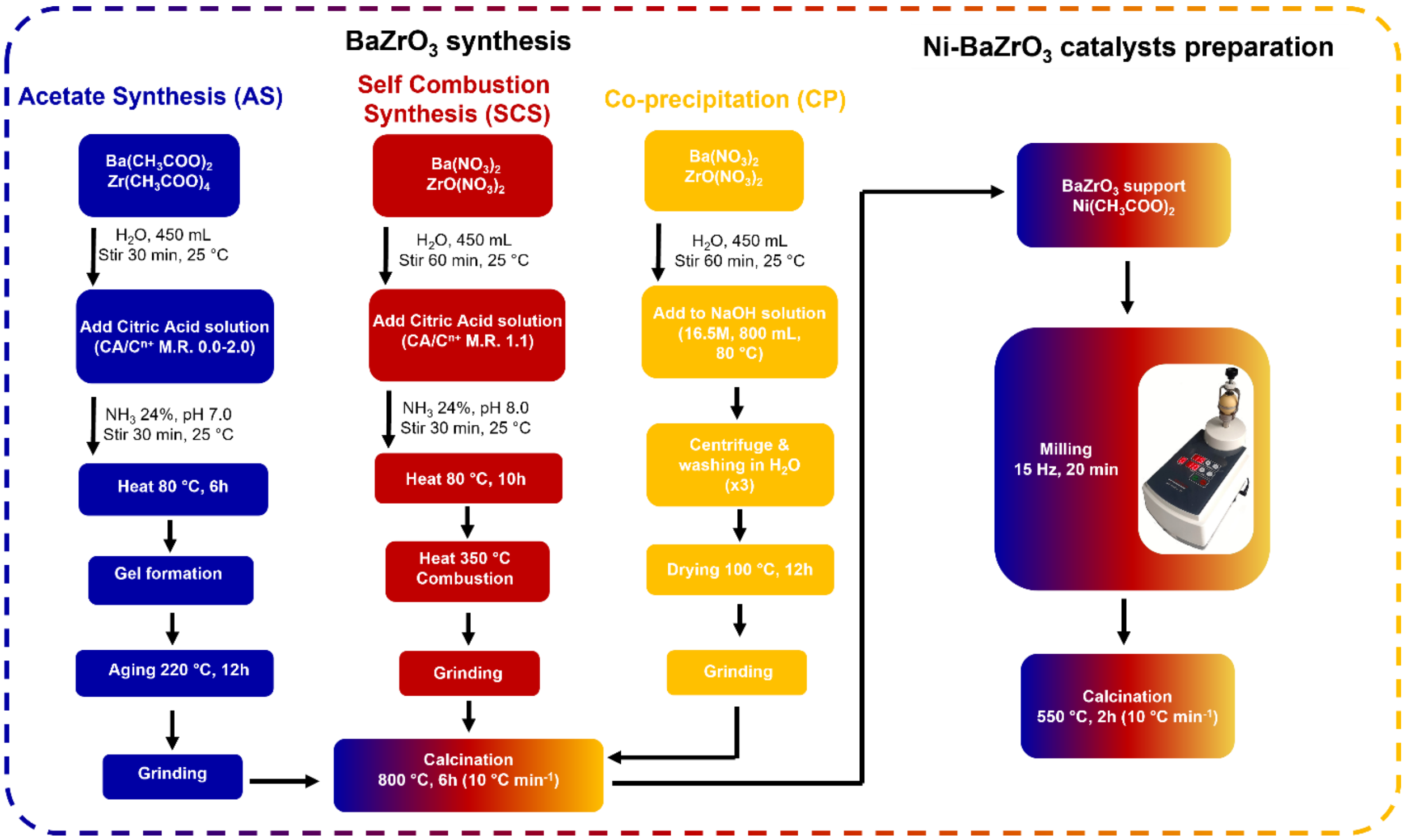

Scheme 1. $BaZrO_3$ preparation via the Acetate Synthesis (AS), the Co-Precipitation route (CP), and the Self Combustion Synthesis (SCS); on the right, the synthesis of 10% Ni-$BaZrO_3$ catalysts via mechanochemical synthesis. CA/C$^+$: citric acid-to-cation ratio.

The acetate precursors dissolution involves the formation of acetic acid in the solution, according to the following reactions:

$$Ba(CH_3COO)_{2\,(s)} + 2H_2O \rightarrow Ba^{2+}{}_{(aq)} + 2C_2H_4O_{2\,(l)} + 2OH^-{}_{(aq)} \quad (S4)$$

$$Zr(CH_3COO)_{4\,(s)} + 4H_2O \rightarrow Zr^{4+}{}_{(aq)} + 4C_2H_4O_2 + 4OH^-{}_{(aq)} \quad (S5)$$

while the decomposition of $Ba(NO_3)_2$ and $ZrO(NO_3)_2$ brings to the formation of nitrate ions:

$$Ba(NO_3)_{2\,(s)} + 2H_2O \rightarrow Ba^{2+}{}_{(aq)} + 2NO_3^-{}_{(aq)} \quad (S6)$$

$$ZrO(NO_3)_{2\,(s)} + 2H_2O \rightarrow ZrO^{2+}{}_{(aq)} + 2NO_3^-{}_{(aq)} \quad (S7)$$

Clearly, the presence of nitrate ions can cause the formation of NO_x species during the evaporation. Also, NH_4OH (which is added to the solution to deprotonate the citric acid) reacts with the nitric acid to form NH_4NO_3: [16,17]

$$NH_4OH_{(l)} + HNO_{3\,(l)} \rightarrow NH_4NO_{3\,(l)} + H_2O \tag{S8}$$

Ammonium nitrate can then decompose during the combustion process: [18,19]

$$NH_4NO_3 + O_2 \rightarrow NO_x + O_2 + H_2O \tag{S9}$$

Since ammonium nitrate is an oxidant agent, its formation is very important when a self-combustion process is carried out and it is then required. On the other hand, the use of nitrates precursor brings to the release of a large amount of NO_x, not only from the decomposition of the salt or its thermal decomposition, but also from the inside reactions during the gelation, aging and combustion steps.

Apparently, the formation of ammonium nitrate during a sol-gel synthesis using acetates is not reported in literature. Instead, ammonium hydroxide and acetic acid could react forming ammonium acetate:

$$NH_4OH_{(l)} + C_2H_4O_{2\,(l)} \rightarrow NH_4CH_3CO_{2\,(s)} + H_2O \tag{S10}$$

which is commonly a solid. There is no evidence of ammonium acetate presence in the acetate synthesis method, but a partial formation is possible. Ammonium acetate has no environmental impact reported. NH_4OH is less reactive when a nitrate source is not present, and consequently it persists during and after the gelation if acetates are used as precursors: it can evaporate during the evaporation and the drying process, and a possible capture of ammonia can be then integrated in the production scale.

The gel formed during the acetate preparation was usually transferred into a muffle and treated at 220 °C for 12 hours. This led to the formation of a dried brown sponge, that was grinded. As it

is possible to observe from **Figure S16**, the resulting sponges had several differences in volume, color, and morphologies. By using $CA/C^+<1$ (**Figure S16a-b**), the sponges are light brown colored, with a low density and with a "coral-like" morphology. For $CA/C^+>1$ (**Figure S16c-d**), they are darker and with a higher density.

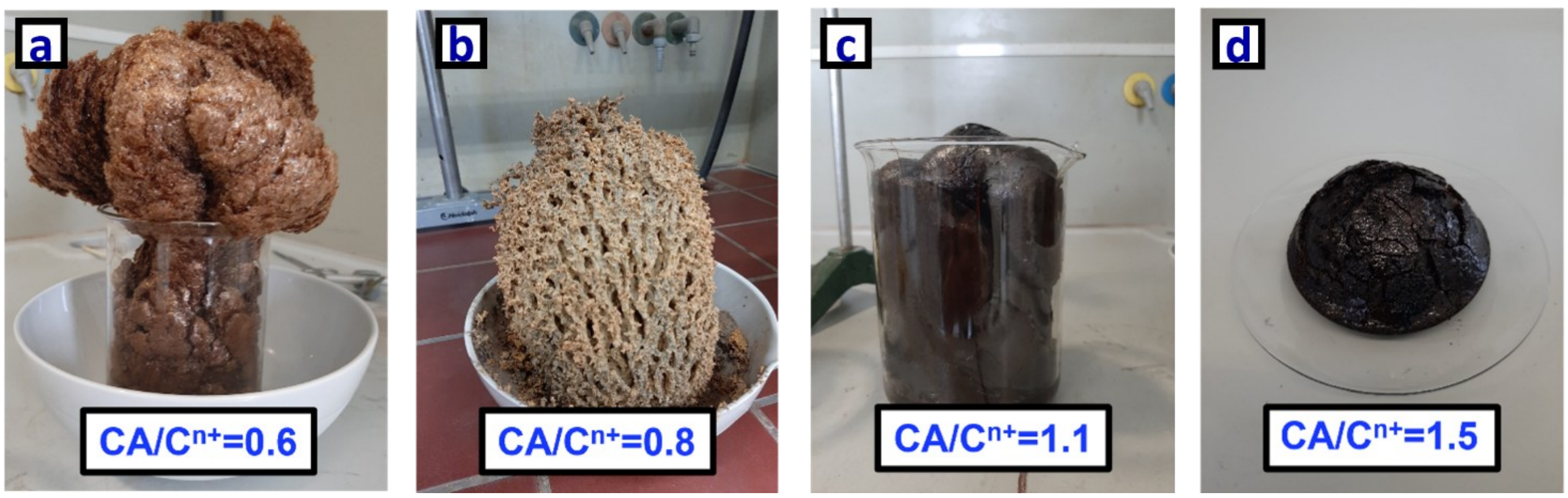

Figure S16. Pictures of the sponges resulting from the aging step at 220 °C for different CA/C^+ molar ratio values.

Since the networking process in sub-stoichiometric conditions is limited by the lack of complexing agent, an open porosity of the gel is expected, and a lot of liquid (in our case, acetic acid from acetate dissolution, unreacted NH_4OH and water) is then entrapped inside the gel (**Figure S16a**). Because of the increase in temperature up to 220 °C, the liquid inside the pores evaporates, and a large amount of gas is then released. Simultaneously, the citrate structure starts to weaken by the partial thermal degradation of the citrate species.[18] These two facts cause a significant increase in volume during the drying, such as for $CA/C^+=0.6$ and $CA/C^+=0.8$ samples (**Figure S16a-b**). In the case of the stochiometric CA/C^+, the porosity tends to be close, and the amount of liquid inside the gel is less and the gas is also entrapped inside the pores (**Figure S16b**). Since the chelation grade is higher than before, the network is more resistant, and this results in a

“panettone-like” sponge (**Figure S16c**). This effect is more evident in excess of citric acid, where the sponge is much denser (**Figure S16d**). The porosity of the sponge, together with the grade of chelation, affects the morphological properties of the resulting perovskites.

The AS is evaluated against SCS and CP in terms of sustainability, safety, cost and scalability, together with the advantages of using ball milling to prepare metal-supported catalysts.

- Acetate Synthesis has a lower environmental impact compared to SCS and CP. This is mainly due to the absence of nitrate salts, which are problematic because their production involves nitric acid[20] and their decomposition releases NO_x.[21,22] In contrast, the thermal decomposition of Ba and Zr acetates in air releases only water and CO_2.[21] Additionally, AS requires no special solvents or chemicals and uses significantly less water than CP. These features make AS more aligned with green chemistry principles and sustainable material production. Finally, citric acid (CA) was used as complexing agent, which is another chemical with a low environmental impact.
- SCS and CP methods present several safety risks. Nitrates are flammable and potentially explosive, requiring strict handling and storage measures, particularly in industrial settings. The SCS process involves also open flames during gel combustion, while CP requires the use of highly concentrated hot NaOH solution to precipitate Ba, creating further safety hazards.[23] These issues can sometimes limit the feasibility of SCS and CP in large-scale production.
- Costs and scalability: the AS method relies on cheap precursors and requires no specific treatments, making it both economically advantageous and easily scalable for industrial production.

- Regarding the deposition of the Ni active phase, mechanochemistry is now widely recognized as a green and sustainable approach for material synthesis,[24] including metal-supported catalysts:[25] it eliminates the need for solvents, saves water and significantly reduces gaseous emissions during processing. By removing solvents, the safety issues of metal-containing solutions handling and storage are also bypassed. Moreover, the related thermal treatments are also drastically reduced, and thus the required energy consumption. Hence, mild-energy ball milling has proven to be a fast and efficient technique for the preparation of metal-supported catalysts.[26,27]

REFERENCES

(1) Danielis, M.; Felli, A.; Zampol, M.; Fonda, N.; Brüner, P.; Grehl, T.; Furlani, E.; Maschio, S.; Colussi, S.; Trovarelli, A. Tuning Chemical and Morphological Properties of Ceria Nanopowders by Mechanochemistry. *ACS Omega* **2024**, *9* (10), 12046–12059. https://doi.org/10.1021/acsomega.3c09926.

(2) De Jesus, J. C.; González, I.; Quevedo, A.; Puerta, T. Thermal Decomposition of Nickel Acetate Tetrahydrate: An Integrated Study by TGA, QMS and XPS Techniques. *J. Mol. Catal. A Chem.* **2005**, 228, 283–291. https://doi.org/10.1016/j.molcata.2004.09.065.

(3) Shi, F.; Dong, H.; Liu, Q.; Yang, J.; Ren, S.; Sun, H.; Xiong, J. Investigation and Theoretical Calculation of the Lattice Vibrational Spectra of BaZrO3 Ceramic. *J. Mater. Sci.: Mater. Electron.* **2017**, *28* (4), 3467–3473. https://doi.org/10.1007/s10854-016-5944-9.

(4) Toulouse, C.; Amoroso, D.; Xin, C.; Veber, P.; Hatnean, M. C.; Balakrishnan, G.; Maglione, M.; Ghosez, P.; Kreisel, J.; Guennou, M. Lattice Dynamics and Raman Spectrum of BaZrO3 Single Crystals. *Phys. Rev. B* **2019**, *100* (13), 134102. https://doi.org/10.1103/PhysRevB.100.134102.

(5) Pasierb, P.; Komornicki, S.; Rokita, M.; Rękas, M. Structural Properties of Li2CO3–BaCO3 System Derived from IR and Raman Spectroscopy. *J. Mol. Struct.* **2001**, *596* (1), 151–156. https://doi.org/10.1016/S0022-2860(01)00703-7.

(6) Shiratori, Y.; Pithan, C.; Dornseiffer, J.; Waser, R. Raman Scattering Studies on Nanocrystalline BaTiO3 Part I - Isolated Particles and Aggregates. *J. Raman Spectrosc.* **2007**, *38* (10), 1288–1299. https://doi.org/10.1002/jrs.1764.

(7) Okura, K.; Miyazaki, K.; Muroyama, H.; Matsui, T.; Eguchi, K. Ammonia Decomposition over Ni Catalysts Supported on Perovskite-Type Oxides for the on-Site Generation of Hydrogen. *RSC Adv.* **2018**, *8* (56), 32102–32110. https://doi.org/10.1039/c8ra06100a.

(8) Chen, Z.; Jiang, B.; Lu, Z.; Huang, J.; Shi, J.; Liu, M. Strong Metal-Support Interaction-Induced Hydrogen Spillover Boosts Thermocatalytic Ammonia Decomposition. *Small* **2025**, 2500830. https://doi.org/10.1002/smll.202500830.

(9) Wang, Y.; Mao, X.; Yang, J.; Wang, J.; Guan, W.; Chen, J.; Han, B.; Tian, Z. One-Step Synthesis of Ni/Yttrium-Doped Barium Zirconates Catalyst for on-Site Hydrogen Production from NH3 Decomposition. *Int. J. Hydrogen Energy* **2022**, *47* (4), 2608–2621. https://doi.org/10.1016/j.ijhydene.2021.10.187.

(10) Ren, H.; Zhao, Y.; Luo, Z.; Hu, E.; Yin, G.; Huang, Z. Experimental and Theoretical Study of BaZrO3 Supported Ru-Ni Catalysts for Ammonia Decomposition Reaction. *Fuel* **2026**, *423*, 139286. https://doi.org/10.1016/j.fuel.2026.139286.

(11) Ali, A.; Ibrahim Aqu, L.; Ajeebi, A. M.; Usman, M.; Siddiqui, M. A. S.; Abdul Aziz, Md.; Nasiruzzaman Shaikh, M. Alkali and Alkaline Earth Metal-Promoted Ni/LaMnO3 Perovskite Catalysts for Efficient Ammonia Decomposition. *Int. J. Hydrogen Energy* **2026**, *219*, 153724. https://doi.org/10.1016/j.ijhydene.2026.153724.

(12) Ali, A.; Ajeebi, A. M.; Usman, M.; Aktary, M.; Hossain, M. M.; Alghamdi, H.; Zahir, M. H.; Shaikh, M. N. LaMnO3 Perovskite Supported Co-Ni Catalysts for Efficient Ammonia Decomposition. *New J. Chem.* **2025**, *49* (33), 14442–14457. https://doi.org/10.1039/d5nj01949d.

(13) Chang, F.; Wu, H.; Pluijm, R. Van Der; Guo, J.; Ngene, P.; De Jongh, P. E. Effect of Pore Confinement of NaNH2 and KNH2 on Hydrogen Generation from Ammonia. *J. Phys. Chem. C* **2019**, *123* (35), 21487–21496. https://doi.org/10.1021/acs.jpcc.9b03878.

(14) Li, Y.; Zhang, W.; Ren, J.; Zhou, W.; Wang, Z. Ammonia Decomposition for Carbon-Free Hydrogen Production over Ni/Al-Ce Catalysts: Synergistic Effect between Al and Ce. *Fuel* **2024**, *358*, 130176. https://doi.org/10.1016/j.fuel.2023.130176.

(15) Yoon, Y.; Choi, Y.; Bae, Y.; Hong, J.; Kim, K. Ni-Based Alloy Catalysts for Cost-Effective Hydrogen Production from Ammonia Decomposition. *Appl. Surf. Sci. Adv.* **2025**, *27*. https://doi.org/10.1016/j.apsadv.2025.100759.

(16) Sutka, A.; Mezinskis, G. Sol-Gel Auto-Combustion Synthesis of Spinel-Type Ferrite Nanomaterials. *Front. Mater. Sci.* **2012**, *6* (2), 128–141. https://doi.org/10.1007/s11706-012-0167-3.

(17) Manju, P.; Babu, A.; Sruthy, S. P.; Tirumalarao, D.; Abhijith, P. V.; Kamble, V. B.; Jaiswal-Nagar, D. Variation of Citrate to Metal Cation Ratio for Dense and Phase Pure BaZrO3 via Autocombustion Synthesis. *J. Am. Ceram, Soc.* **2022**, *105* (8), 5082–5101. https://doi.org/10.1111/jace.18477.

(18) Babu, A.; Tirumalarao, D.; Das, S.; Dixit, V.; Sruthy, S. P.; Vijayan, V.; Jaiswal-Nagar, D. Effect of PH Variation on Citrate Nitrate Sol-Gels Obtained from Auto-Combustion Method: Synthesis, Calculations and Characterisations of Extremely Dense BaZrO3 Ceramic. *Open Ceram.* **2022**, *12*, 100303. https://doi.org/10.1016/j.oceram.2022.100303.

(19) Chaturvedi, S.; Dave, P. N. Review on Thermal Decomposition of Ammonium Nitrate. *J. Energ. Mater.* **2013**, *31* (1), 1–26. https://doi.org/10.1080/07370652.2011.573523.

(20) Bernauer, M.; Bernauer, B.; Sádovská, G.; Sobalík, Z. High-Temperature Decomposition of N2O from the HNO3 Production: Process Feasibility Using a Structured Catalyst. *Chem. Eng. Sci.* **2020**, *220*, 115624. https://doi.org/10.1016/j.ces.2020.115624.

(21) Marceau, E.; Che, M.; Čejka, J.; Zukal, A. Nickel(II) Nitrate vs. Acetate: Influence of the Precursor on the Structure and Reducibility of Ni/MCM-41 and Ni/Al-MCM-41 Catalysts. *ChemCatChem* **2010**, *2* (4), 413–422. https://doi.org/10.1002/cctc.200900289.

(22) Sietsma, J. R. A.; Meeldijk, J. D.; Versluijs-Helder, M.; Broersma, A.; Jos Van Dillen, A.; De Jongh, P. E.; De Jong, K. P. Ordered Mesoporous Silica to Study the Preparation of Ni/SiO2 Ex Nitrate Catalysts: Impregnation, Drying, and Thermal Treatments. *Chem. Mater.* **2008**, *20* (9), 2921–2931. https://doi.org/10.1021/cm702610h.

(23) Boschini, F.; Rulmont, A.; Cloots, R.; Vertruyen, B. Rapid Synthesis of Submicron Crystalline Barium Zirconate BaZrO3 by Precipitation in Aqueous Basic Solution below 100 °C. *J. Eur. Ceram. Soc.* **2009**, *29* (8), 1457–1462. https://doi.org/10.1016/j.jeurceramsoc.2008.09.001.

(24) Ardila-Fierro, K. J.; Hernández, J. G. Sustainability Assessment of Mechanochemistry by Using the Twelve Principles of Green Chemistry. *ChemSusChem*. **2021**, *14* (10) 2145–2162. https://doi.org/10.1002/cssc.202100478.

(25) Danielis, M.; Colussi, S.; Divins, N. J.; Soler, L.; Trovarelli, A.; Llorca, J. Mechanochemistry: A Green and Fast Method to Prepare a New Generation of Metal Supported Catalysts. *Johns. Matthey Technol. Rev.* **2024**, *68* (2), 217–231. https://doi.org/10.1595/205651323X16933888986996.

(26) Colacino, E.; Isoni, V.; Crawford, D.; García, F. Upscaling Mechanochemistry: Challenges and Opportunities for Sustainable Industry. *Trends Chem.* **2021**, *3* (5), 335–339. https://doi.org/10.1016/j.trechm.2021.02.008.

(27) Muñoz-Batista, M. J.; Rodriguez-Padron, D.; Puente-Santiago, A. R.; Luque, R. Mechanochemistry: Toward Sustainable Design of Advanced Nanomaterials for Electrochemical Energy Storage and Catalytic Applications. *ACS Sustain. Chem. Eng.* **2018**, *6* (8), 9530–9544. https://doi.org/10.1021/acssuschemeng.8b01716.
